# Unraveling the Mechanism of the Photodeprotection Reaction of 8-Bromo- and 8-Chloro-7-hydroxyquinoline Caged Acetates

**DOI:** 10.1002/chem.201200366

**Published:** 2012-04-18

**Authors:** Jiani Ma, Adam C Rea, Huiying An, Chensheng Ma, Xiangguo Guan, Ming-De Li, Tao Su, Chi Shun Yeung, Kyle T Harris, Yue Zhu, Jameil L Nganga, Olesya D Fedoryak, Timothy M Dore, David Lee Phillips

**Affiliations:** a Department of Chemistry, The University of Hong KongPokfulam Road, Hong Kong S.A.R. (P.R. China), Fax: (+852) 2957-1586; b Department of Chemistry, University of GeorgiaAthens, Georgia 30602-2556 (USA), Fax: (+1) 706-542-9454

**Keywords:** cage compounds, photodeprotection, proton transfer, quinoline, time-resolved spectroscopy

## Abstract

Photoremovable protecting groups (PPGs) when conjugated to biological effectors forming “caged compounds” are a powerful means to regulate the action of physiologically active messengers in vivo through 1-photon excitation (1PE) and 2-photon excitation (2PE). Understanding the photodeprotection mechanism is important for their physiological use. We compared the quantum efficiencies and product outcomes in different solvent and pH conditions for the photolysis reactions of (8-chloro-7-hydroxyquinolin-2-yl)methyl acetate (CHQ-OAc) and (8-bromo-7-hydroxyquinolin-2-yl)methyl acetate (BHQ-OAc), representatives of the quinoline class of phototriggers for biological use, and conducted nanosecond time-resolved spectroscopic studies using transient emission (ns-EM), transient absorption (ns-TA), transient resonance Raman (ns-TR^2^), and time-resolved resonance Raman (ns-TR^3^) spectroscopies. The results indicate differences in the photochemical mechanisms and product outcomes, and reveal that the triplet excited state is most likely on the pathway to the product and that dehalogenation competes with release of acetate from BHQ-OAc, but not CHQ-OAc. A high fluorescence quantum yield and a more efficient excited-state proton transfer (ESPT) in CHQ-OAc compared to BHQ-OAc explain the lower quantum efficiency of CHQ-OAc relative to BHQ-OAc.

## Introduction

Photoremovable protecting groups (PPGs) have become powerful tools for probing biological systems through their conjugation to biological effectors, forming “caged compounds”.^1^ The photorelease reactions of PPGs are mostly initiated by one-photon excitation of ultraviolet (UV) or near-UV light. Recently, there has been increasing interest in developing PPGs that can be excited by two-photon excitation (2PE) because of their special advantages (such as minimizing damage to the biological system by using lower energy photons in the near infrared region and improved spatial resolution) for the release of biological effectors in biological systems and one can refer to two very recent reviews for details.^2^ Only a few PPGs contain chromophores with large enough two photon excitation cross sections that can be used effectively in physiology experiments; therefore, there are relatively few classes of PPGs that have been developed for 2PE compared with those developed for 1PE excitation.

**Scheme 1:**
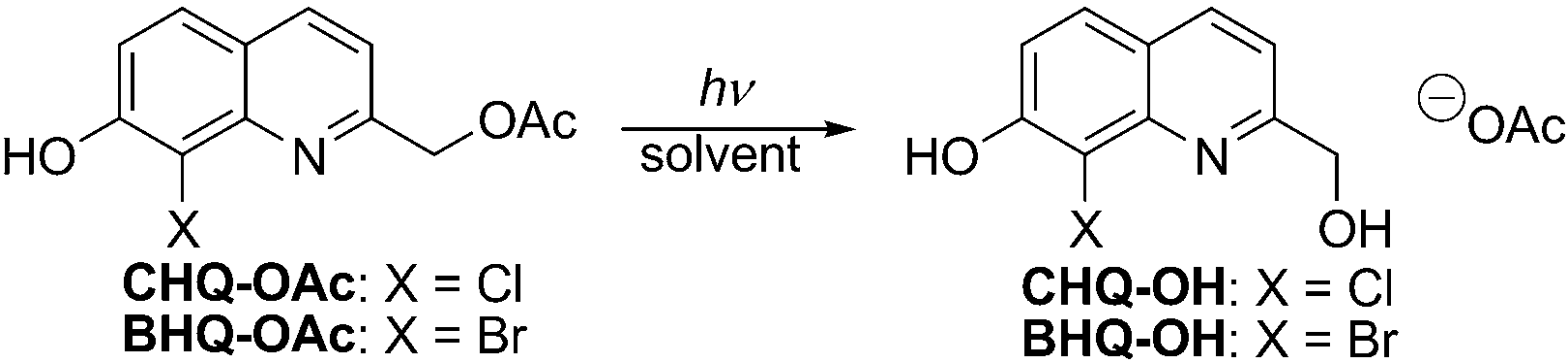
Photolysis of CHQ-OAc and BHQ-OAc.

Dore and co-workers discovered that the quinoline-based phototriggers have promise in mediating the release of physiologically active messengers through both one-photon excitation (1PE) and 2PE.^3^ (8-Chloro-7-hydroxyquinolin-2-yl)methyl acetate (CHQ-OAc) and (8-bromo-7-hydroxyquinolin-2-yl)methyl acetate (BHQ-OAc) photolyze in response to irradiation with ultraviolet (1PE) or near-visible light (2PE), releasing acetate and the remnant of the protecting group 8-chloro-2-(hydroxymethyl)quinolin-7-ol (CHQ-OH) and 8-bromo-2-(hydroxymethyl)quinolin-7-ol (BHQ-OH), respectively (Scheme 1). BHQ in particular has been reported to be a good phototrigger for mediating the release of biologically relevant effectors in vivo through 2PE. It has been used to pinpoint the timing and location of morpholino activation in zebrafish with 2PE,^4^ regulate the action of a thrombin aptamer (HD1),^5^ and initiate the expression of GFP.^6^ In response to these successful applications, it is important to characterize and understand the deprotection reaction mechanism, so that biological experiments can be conducted free of doubt about the performance of the phototrigger, and improvements to the design of quinoline-based protecting groups can be made.

According to initial investigations, the photodeprotection reaction proceeds through a singlet transient species and the release of the protected group likely takes place through a solvent-assisted photoheterolysis (S_N_1) reaction mechanism.3b This conclusion was based on indirect information such as an oxygen quenching experiment conducted in acetonitrile (MeCN), rather than in water-containing solutions. Recent work has shown that BHQ-OAc and CHQ-OAc exhibit totally different behavior in water-containing systems compared with their behavior in inert organic solvents like MeCN.^7^ The 1PE quantum efficiency (*Q*_u_) and the 2PE uncaging action cross-section (*δ*_u_) for the photolysis of CHQ-OAc was found to be noticeably lower than that for BHQ-OAc (*Q*_u_=0.29 for BHQ-OAc3a and 0.10 for CHQ-OAc;3c δ_u_=0.59 GM for BHQ-OAc3a and 0.12 GM for CHQ-OAc3c). If a singlet state transient species acts as the precursor for the deprotection reaction, the *Q*_u_ of CHQ-OAc could be expected to be significantly higher than that of BHQ-OAc since the introduction of the heavy atom bromine into its structure would better facilitate a competing intersystem crossing (ISC) process in BHQ-OAc, and the depletion of the singlet state would be more efficient in the bromine containing system than the chlorine containing compound. In addition, the excited state proton transfer (ESPT) process, which has been extensively studied for the parent compound 7-hydroxyquinoline (7-HQ),^8^ was not examined in detail for BHQ-OAc, and the role of ESPT in the water-containing solutions has not yet been examined. In view of these issues, it is desirable to explicitly investigate the reaction mechanism of BHQ-OAc in neutral aqueous solutions not only to help gain a better understanding of the reaction mechanism, but also to acquire information that may assist in the design of improved two-photon phototrigger compounds.

To help resolve these issues, we examined the photolysis reactions of BHQ-OAc and CHQ-OAc to determine the quantum efficiency (*Q*_u_) and initial product outcome under different solvent and pH conditions, and we conducted comparative nanosecond time-resolved spectroscopic studies using transient emission (ns-EM), transient absorption (ns-TA), transient resonance Raman (ns-TR^2^), and time-resolved resonance Raman (ns-TR^3^) spectroscopies. Density functional theory (DFT) calculations were utilized to help make assignments of the experimental Raman spectra to probable intermediate species and to study the solvolysis reaction that follows the deprotection reaction.

## Results and Discussion

**Scheme 2:**
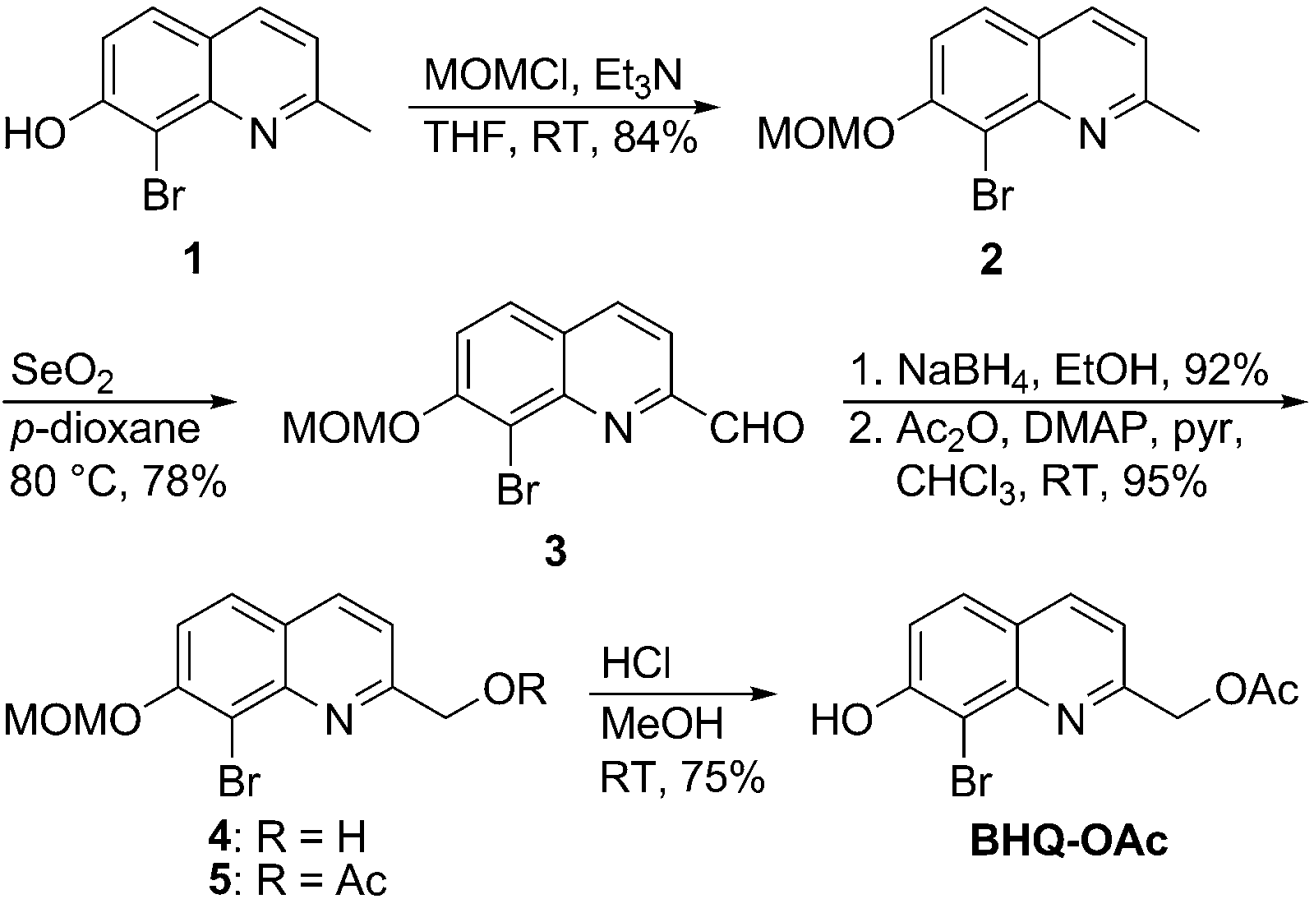
Preparation of BHQ-OAc.

**Synthesis**: BHQ-OAc was synthesized from bromoquinoline **1**3b by the route shown in Scheme 2, which is a modification of the route previously reported.3a A methoxymethyl (MOM) ether was used as a protecting group instead of a *tert*-butyldiphenylsilyl (TBDPS) group, and the bromine was carried through the sequence instead of being added near the end of the synthesis. This alternative synthesis of BHQ-protected acetate represents an improvement over the originally reported route.3a It is high yielding and eliminates the use of an expensive *tert*-butyldiphenylsilyl protecting group. This new route will be useful when protecting other molecules with carboxylate and other functional groups. CHQ-OAc was synthesized as previously described in the literature.3c Authentic samples of the photolysis products BHQ-OH and CHQ-OH were synthesized from the corresponding acetates as previously described for BHQ-OH.3a

**Solvent effects on quantum efficiency**: The quantum efficiencies (*Q*_u_) for the photolyses of BHQ-OAc and CHQ-OAc at 254 nm in each of the three solvents were determined by methods previously described (Table 1).3b, 9 Photolysis reactions were conducted at 254 nm, rather than near the *λ*_max_ value of 369 nm to more closely match the time-resolved studies, which used 266 nm for excitation (see below). The value of *Q*_u_ was lower for CHQ-OAc than that for BHQ-OAc. At low pH, *Q*_u_ for CHQ-OAc is relatively unchanged compared to the value measured in the neutral mixed H_2_O/MeCN solvent, but *Q*_u_ for BHQ-OAc is lower under acidic conditions compared with a neutral environment.

**Table 1:** Quantum efficiencies of BHQ-OAc and CHQ-OAc photolyses.

Chromophore	Solvent	*Q*_u_ at 254 nm
BHQ-OAc	60:40 H_2_O/MeCN, pH 7	0.25
	60:40 H_2_O/MeCN, pH 4	0.17
	KMOPS buffer, pH 7.2	0.26
CHQ-OAc	60:40 H_2_O/MeCN, pH 7	0.09
	60:40 H_2_O/MeCN, pH 4	0.10
	KMOPS buffer, pH 7.2	0.14

The values of *Q*_u_ at 254 nm in the two neutral solvents are similar to those reported for BHQ-OAc (0.29)3a and CHQ-OAc (0.10)3c at 365 nm in KMOPS buffer. The lower *Q*_u_ for CHQ-OAc compared with BHQ-OAc and the previous observation that CHQ-OAc is slightly more fluorescent (*λ*_ex_=365 nm,) than BHQ-OAc3c is inconsistent with time-resolved infrared spectroscopy (TRIR) and Stern–Volmer quenching experiments, which indicated that BHQ-OAc photolyzed through a singlet excited state.3b If the reaction proceeded through a singlet excited state, the quantum efficiency for CHQ-OAc should be higher than that for BHQ-OAc, because the bromine would promote intersystem crossing (ISC), which would deplete the singlet excited state of BHQ-OAc. Chlorine does not significantly promote ISC.

**Figure 1:**
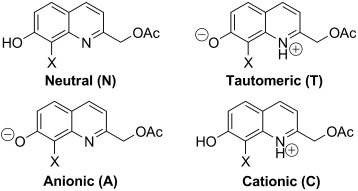
Prototropic states of XHQ-OAc.

The p*K*_a_ of both BHQ-OAc and CHQ-OAc is 6.8, and the UV/Vis absorbance spectra of BHQ-OAc and CHQ-OAc in KMOPS are nearly identical.3a, 7c In solution, 8-substituted-7-hydroxyquinolines can exist as a single prototropic form or a mixture of several (Figure 1). At neutral pH in 60:40 H_2_O/MeCN, resonance Raman spectroscopy revealed that BHQ-OAc and CHQ-OAc exist mainly as the neutral (**N**) prototropic form with some contribution of the anionic (**A**) form, but CHQ-OAc has a larger amount of the tautomeric (**T**) form present.7a, c At low pH, less of the anionic (**A**) form and more of the neutral (**N**) and cationic (**C**) forms are expected to be present, which are the least absorptive forms of the quinoline and would diminish *Q*_u_, as we observed for BHQ-OAc. It is not clear why *Q*_u_ values at neutral and acidic pH are similar for CHQ-OAc.

**Product outcome studies in different solvents**: The initial products formed in each photolysis reaction were determined by HPLC and MS analysis. Samples of BHQ-OAc and CHQ-OAc in KMOPS buffer were irradiated at 254 nm in a photoreactor to determine how much exposure time was required to generate amounts of the initially formed products of the reaction that could be observed by the UV/Vis detector on the HPLC and ESI-MS analysis of the eluate from the column. BHQ-OAc required only 15 s of irradiation, whereas CHQ-OAc needed 30 s. This is consistent with the relative sensitivities to 1PE (ɛ×*Q*_u_) of the chromophores: 754 for BHQ-OAc3a versus 280 for CHQ-OAc.3c CHQ-OAc is less sensitive to photolysis than BHQ-OAc.

The product outcome of the photolysis of CHQ-OAc under different solvent conditions was determined by irradiating 100 μm samples of CHQ-OAc in 60:40 H_2_O/MeCN pH 4, 60:40 H_2_O/MeCN pH 7, KMOPS buffer pH 7.2, and dry MeCN and analyzing the reaction by HPLC. Photolysis in a high pH environment (e.g., 60:40 H_2_O/MeCN pH 10) was not possible because spontaneous hydrolysis of the acetate at pH 10 was sufficiently rapid to compete with photolysis. Irradiation of CHQ-OAc in dry MeCN did not lead to observable photolysis products, even after several minutes. If irradiation was extended to 45 min or longer, the starting material began to degrade and some new peaks were observed by HPLC, but we could not identify the compounds giving rise to these new peaks.

**Figure 2:**
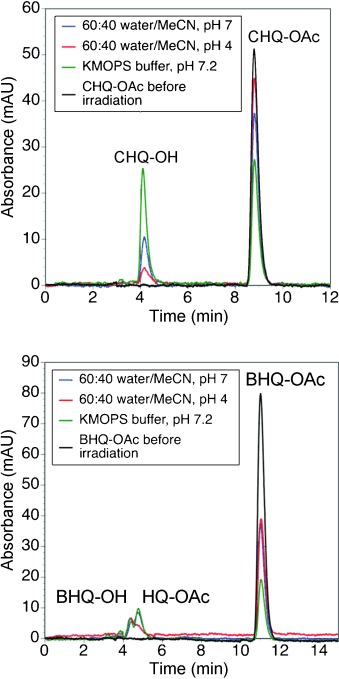
HPLC chromatograms of the photolysis of a) CHQ-OAc (*t*_R_=8.8 min) to CHQ-OH (*t*_R_=4.1 min) and b) BHQ-OAc (*t*_R_=11.0 min) to BHQ-OH (*t*_R_=4.4 min) and HQ-OAc (*t*_R_=4.8 min).

In each aqueous solvent, a single new peak was observed at *t*_R_=4.1 min in the HPLC chromatogram (Figure 2 a). ESI-MS analysis of the eluate corresponding to that peak produced a signal at *m*/*z* 210, which corresponds to MH^+^ of CHQ-OH. This assignment was confirmed by conducting the same analysis on an authentic sample of CHQ-OH. Inspection of the chromatograms in Figure 2 a reveals that the extent of disappearance of CHQ-OAc is greatest in aqueous KMOPS buffer. The photolysis is less efficient in the mixed H_2_O/MeCN solvents, and the reaction proceeds to the least extent after 30 s in 60:40 H_2_O/MeCN pH 4 in which the less absorbent neutral (**N**) and cationic (**C**) forms of CHQ-OAc predominate. The relative photolysis efficiencies are consistent with the distribution of prototropic ground states of CHQ-OAc in neutral and basic water-rich solutions and MeCN.7c

The photolysis of BHQ-OAc in the four solvents was more complex than that of CHQ-OAc. Like CHQ-OAc, BHQ-OAc did not photolyze in the dry MeCN solvent system, and BHQ-OAc disappeared to the greatest extent in KMOPS buffer and to the least extent in acidic media. In the three water-rich solutions, HPLC analysis of the photolysis reaction revealed that two different photolysis products were formed with *t*_R_=4.4 and 4.8 min (Figure 2 b), and ESI-MS of the eluate corresponding to each peak showed signals at *m*/*z* 254/256 and 218, respectively. The retention time of 4.4 min corresponds to that of an authentic sample of BHQ-OH, and ESI-MS of BHQ-OH presents two peaks equal in magnitude for MH^+^ at *m*/*z* 254 and 256, as a result of the isotopic abundance of ^79^Br and ^81^Br.

**Scheme 3:**
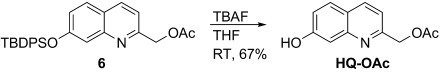
Preparation of HQ-OAc.

Other work has suggested that photoinduced debromination of BHQ competes with the photolysis reaction,7b so we synthesized a sample of the debrominated product (7-hydroxyquinolin-2-yl)methyl acetate (HQ-OAc) from TBDPS-protected quinoline **6**3a (Scheme 3). Analysis of this authentic sample of HQ-OAc revealed that the peak at *t*_R_=4.8 min with *m*/*z* 218 for MH^+^ corresponds to HQ-OAc. Debromination appears to compete with photolysis of the acetate. At first glance, this observation might preclude the use of BHQ as a caging group for biological applications, but yields of released products are consistently 60–70 %.3b HQ-OAc is also photoactive and releases acetate upon exposure to light. These results indicate that the mechanism of light-mediated carboxylate release is complex, but it does not diminish the potential for the use of quinoline-based protecting groups in biological systems.

**Figure 3:**
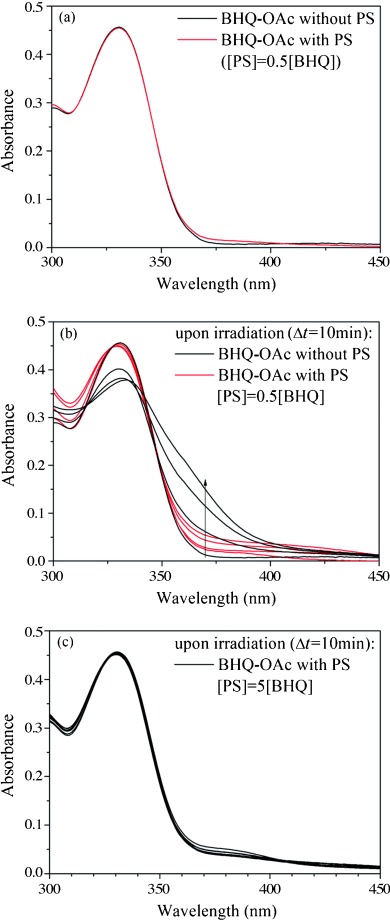
a) Absorption spectra of BHQ-OAc with PS ([PS]≍5×10^−5^ m, [BHQ-OAc]≍1×10^−4^ m) and without PS in H_2_O/MeCN ([BHQ-OAc]≍1×10^−4^ m, 3:2, *v*/*v*, pH 6–7) before irradiation, b) comparison of absorption spectra of BHQ-OAc without PS to those with 0.5 times [PS] upon photolysis in H_2_O/MeCN (3:2, *v*/*v*, pH 6–7), and c) absorption spectra of BHQ-OAc with 5 times [PS] ([PS]≍5×10^−4^ m, [BHQ-OAc]≍1×10^−4^ m) in H_2_O/MeCN (3:2, *v*/*v*, pH 6–7).

**Quenching experiments in a mixed aqueous solvent**: To investigate whether a singlet or triplet species is correlated with the deprotection reaction, quenching experiments were conducted employing the well-known triplet quenching agent potassium sorbate (PS). We compared the UV/Vis absorption spectra of BHQ-OAc before and after irradiation under 266 nm wavelength laser pulses with different concentrations of PS in a neutral mixed aqueous solution (Figure 3).

The absorbance of BHQ-OAc at longer wavelengths (>300 nm) will be focused on here because PS has strong absorption below 300 nm. Before irradiation, the spectra of the solutions without PS and with half of the concentration of PS as BHQ-OAc in the same solution showed analogous features in the region above 300 nm. Photolysis gave rise to an obvious increase of the approximately 370 nm absorbance that is assigned to the absorption of the converted photoproducts in both solutions, and this process was much slower in the solution with PS than in the one without PS. Increasing the concentration of PS in the solution, the absorption of the mixed aqueous solution showed no discernible increase in the 370 nm region from 0 to 30 min. Inhibition of the photochemical reaction by the excess PS suggests a triplet species (e.g., **A**(T_1_) or other triplet intermediates) serves as the precursor of the photochemical reactions of BHQ-OAc in neutral aqueous solutions.

**Figure 4:**
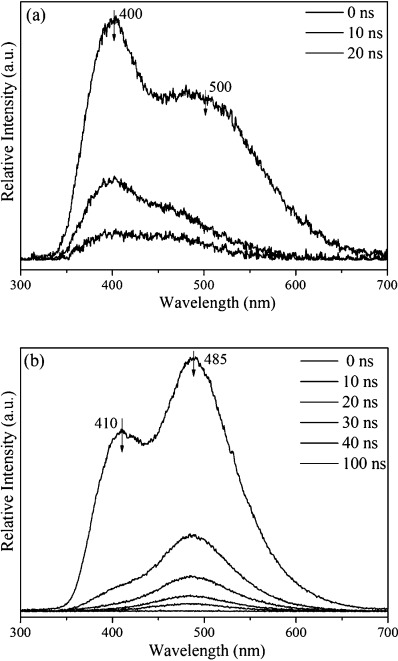
The ns-EM spectra of a) BHQ-OAc and b) CHQ-OAc acquired in H_2_O/MeCN (3:2, *v*/*v*, pH 6–7).

**Nanosecond transient emission spectra (ns-EM)**: Based on steady-state studies of BHQ-OAc,7b the peak in the ns-EM spectra in neutral H_2_O/MeCN (Figure 4 a) with an emission wavelength at approximately 400 nm can be assigned to **N**(S_1_) and the one at approximately 500 nm as **T**(S_1_). **N**(S_1_) is the exclusive species observed in MeCN (Figure 1 S in the Supporting Information), whereas both **N**(S_1_) and **T**(S_1_) are generated in appreciable amounts from excitation in neutral aqueous solutions. Since the steady-state studies demonstrated that the tautomerization of BHQ-OAc is much less efficient compared with 7-HQ, and the population of **T**(S_0_) of BHQ-OAc is rather limited,7a, b any **T**(S_1_) that contributes to the ns-EM spectra was likely almost exclusively produced via an ESPT process.

The ns-EM spectra of CHQ-OAc in neutral H_2_O/MeCN (Figure 4 b) show two species were detected around 410 and 485 nm that were respectively assigned as **N**(S_1_) and **T**(S_1_) of CHQ-OAc. In so far as the fluorescence quantum yield ratios for the **T**(S_1_) to **N**(S_1_) species for the two compounds BHQ-OAc and CHQ-OAc are similar, the different ratios of fluorescence intensities of the approximately 410 and 585 nm bands could indicate that there is a higher concentration of the **T**(S_1_) to **N**(S_1_) species for CHQ-OAc than for BHQ-OAc and hence the ESPT process is favored in CHQ-OAc compared with BHQ-OAc. Also, the lifetimes of both these species of CHQ-OAc are longer than those of BHQ-OAc, suggesting that the singlet excited state faces less competition from other routes, especially the ISC process for the **T**(S_1_) and **N**(S_1_) species of CHQ-OAc. Furthermore, the dual fluorescence quantum yield of CHQ-OAc was about 3 times higher than that of BHQ-OAc, consistent with a previous study that reported CHQ-OAc has a stronger emission than BHQ-OAc,3c which suggests that a larger ratio of singlet-state energy was lost through fluorescence rather than promoting a photochemical reaction. Taken together, these results imply that the ISC process is more efficient for BHQ-OAc, and the detection of **T**(S_1_) demonstrates the occurrence of an ESPT process for BHQ-OAc in water-containing solutions.

**Figure 5:**
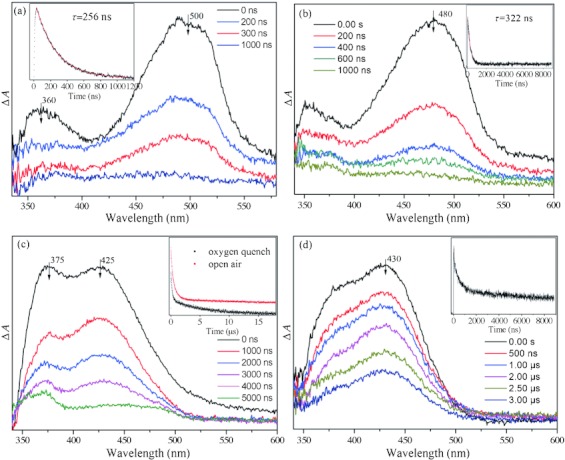
Transient absorption spectra of a) BHQ-OAc and b) CHQ-OAc in MeCN following 266 nm laser irradiation (insets are the time evolution with an initial growth followed by a decay of the Δ*A* monitored at 520 and 480 nm, respectively) and transient absorption spectra of c) BHQ-OAc and d) CHQ-OAc in H_2_O/MeCN (3:2, *v*/*v*, pH 6–7) following 266 nm laser irradiation (insets are the time evolution with an initial growth followed by a decay of the Δ*A* monitored at 370 nm in open air and oxygen-saturated conditions for BHQ-OAc and the time evolution with an initial growth followed by a decay of the Δ*A* for CHQ-OAc monitored at 430 nm).

**Nanosecond transient absorption spectra (ns-TA)**: Upon laser excitation of BHQ-OAc in MeCN, the emission band at 360 nm and a broad band around 500 nm appeared and then decayed to zero (Figure 5 a). Likewise, for CHQ-OAc the ns-TA spectrum showed two peaks at 410 and 485 nm (Figure 5 b). The similar lifetime constants for decay (*τ*=256 and 322 ns) of BHQ-OAc and CHQ-OAc, respectively (insets of Figure 5 a and 5b) with first-order kinetics at each wavelength suggests the two bands result from one species.

In light of the steady-state studies suggesting **N**(S_0_) of BHQ-OAc is the predominant species7a, b and the ns-EM spectra showing **N**(S_1_) is the only species observed in MeCN solution, we propose that **N**(T_1_) is the species observed in the ns-TA spectra obtained in MeCN. The **N**(T_1_) species arises from conversion of **N**(S_1_) through ISC. The decay time of **N**(T_1_) detected here is shorter than a previous time-resolved infrared absorption (TRIR) measurement (*τ*=590 ns, *k*=1.7×10^6^ s^−1^).3b A plausible explanation for this difference stems from the different experimental conditions: the ns-TA experiment here was performed in air-saturated MeCN, whereas the TRIR study was conducted in argon-saturated MeCN. The shorter decay time constant measured under the air-saturated MeCN condition (256 ns) compared with the argon-saturated MeCN condition (580 ns) is consistent with the triplet assignment for the observed transient species. To further verify, ns-TA experiments were also conducted in oxygen-saturated MeCN (Figure 2 S in the Supporting Information) and a decay time of 93 ns was measured. The shortened lifetime of **N**(T_1_) in oxygen-rich conditions corroborates the hypothesis that the species observed in MeCN has a triplet character.

The ns-TA experiments on BHQ-OAc in neutral mixed solvent provided different results (Figure 5 c). The observed Δ*A* of the two bands at 375 and 425 nm did not decay to the baseline as time progressed, indicating that at least two species contribute to the absorption spectra and some species has (have) a long lifetime that might result from the final solvolysis product. Because debromination competes with the acetate deprotection for BHQ-OAc in aqueous solutions, it is necessary to discuss which species is (are) contributing to the long-lived absorption. Dehalogenation does not take place for CHQ-OAc under the same reaction conditions, so the long-lived absorption under irradiation in CHQ-OAc aqueous solution was probably the product generated from the deprotection of the acetate (Figure 5 d). The ns-TA spectra for BHQ-OH, the final product from the deprotection reaction of BHQ-OAc, in H_2_O/MeCN (3:2, pH 7) (Figure 3 S in the Supporting Information) shows close similarity with the spectra seen at later times for the ns-TA spectra of BHQ-OAc in the same solvent. Hence, the species appearing at later times in the ns-TA spectra of BHQ-OAc is probably the final product BHQ-OH. This result further supports the idea that the deprotection reaction takes place in neutral water-containing solutions. The different band shapes in Figure 5 c compared with those in Figure 5 a suggest that **N**(T_1_) was not detected in the H_2_O/MeCN (3:2, pH 7) solution. The ns-TA spectra acquired in an oxygen-saturated H_2_O/MeCN (3:2, pH 7) solution shows the Δ*A* decreases almost to the baseline (Figure 5 c inset). The observed disappearance of BHQ-OH under oxygen-rich conditions provides evidence that a triplet species is needed for the deprotection reaction.

**Figure 6:**
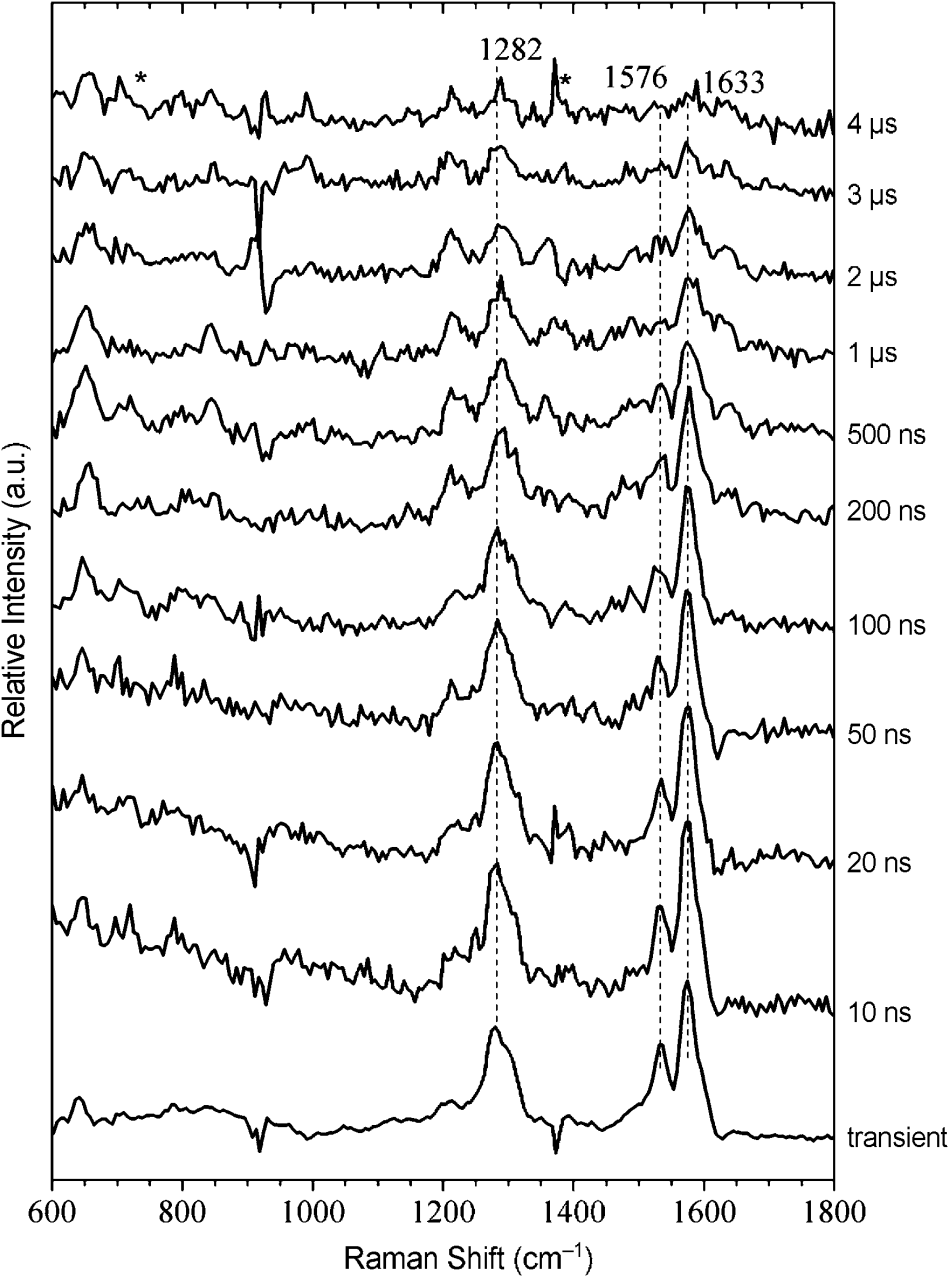
The ns-TR^2^ spectrum (labeled “transient”) and ns-TR^3^ spectra of BHQ-OAc acquired at varying time delays with the 266 nm/320 nm pump/probe wavelengths in MeCN.

**Nanosecond transient resonance Raman (ns-TR^2^) and nanosecond time-resolved resonance Raman (ns-TR^3^) experiments**: The ns-TR^2^ and ns-TR^3^ spectra of BHQ-OAc in MeCN (Figure 6) revealed that the transient species probed in the ns-TR^2^ and ns-TR^3^ experiments are essentially the same with Raman bands around 642, 1282, 1576, and 1633 cm^−1^. Given the same time resolution on the nanosecond scale for the ns-TR^2^ and ns-TR^3^ spectra with that of the ns-EM and ns-TA spectra, it is reasonable to propose **N**(T_1_) is the transient species detected here. The major bands at 1576 and 1633 cm^−1^ show good agreement with the IR bands observed at 1574 and 1632 cm^−1^ for **N**(T_1_) in MeCN.3b The lifetime of the species that was observed in the first 10 ns and subsequently decays on the hundreds of nanosecond timescales measured by the ns-TR^3^ spectra also agrees well with the ns-TA and TRIR results obtained in MeCN.3b The good correlation of the vibrational frequencies and the decay lifetime of this species strongly support that **N**(T_1_) is the intermediate species observed in MeCN. The extra bands at later times around 652, 852, 1214, and 1633 cm^−1^ arise from species formed due to residual water in MeCN.3b

**Figure 7:**
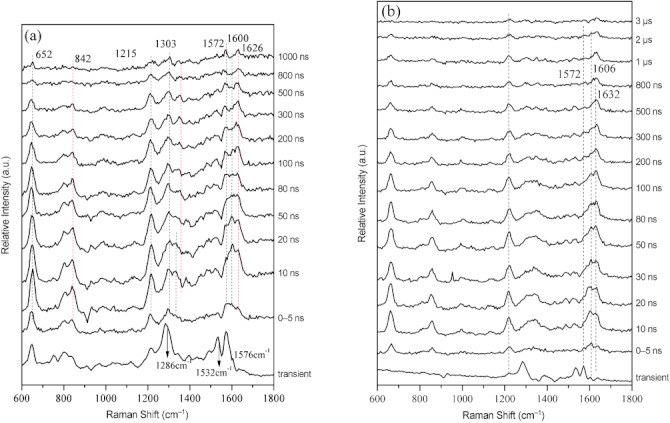
The ns-TR^2^ spectra (labeled “transient”) and ns-TR^3^ spectra of a) BHQ-OAc and b) CHQ-OAc in H_2_O/MeCN (3:2, *v*/*v*, pH 6–7) solution with varying time delays indicated next to the spectra with the 266 nm pump and 320 nm or 355 nm probe laser pulses for BHQ-OAc and CHQ-OAc, respectively.

Unlike the observation in MeCN that the earlier species in the ns-TR^3^ spectra is the same as that observed in the ns-TR^2^ spectrum, the ns-TR^2^ spectrum and ns-TR^3^ spectra (Figure 7) at early time delays are significantly different for BHQ-OAc and CHQ-OAc in neutral H_2_O/MeCN (3:2, *v*/*v*, pH 6–7). This indicates that different photochemical reactions occur after photolysis in neutral aqueous system as compared with MeCN solvent. For BHQ-OAc, two sets of Raman bands are observed within 10 ns in the ns-TR^3^ spectra (indicated with black and red lines, respectively, in Figure 7 a) and appear to decay on different timescales, indicating that they belong to different transient species created after photolysis. However, the complicated TR^3^ spectra with partially overlapping bands from different transient species make the assignments challenging to unravel. Similar result was observed in the spectra of CHQ-OAc (Figure 7 b).

**Scheme 4:**
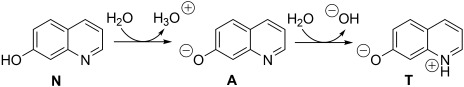
ESPT process of 7-HQ in water.

Because the **T**(S_1_) species of BHQ-OAc and CHQ-OAc were detected in neutral aqueous solution by the ns-EM spectra, it is necessary to take the ESPT reaction into consideration. Previous studies on 3-, 6-, and 7-hydroxyquinoline^10^ reported that the ESPT process for these compounds takes place by first forming the anionic intermediate form **A**(S_1_) (Scheme 4).^[8^ ^g]^ Since **A**(S_1_) was speculated to be the precursor for the deprotection reaction in a previous study3b (although the multiplet of the precursor is now suggested in the present work to be a triplet state), it would be helpful to examine the situation for BHQ-OAc and CHQ-OAc in alkaline solution.

The ns-TR^3^ experiments for photolysis of BHQ-OAc in alkaline aqueous solution (pH 11–12), where the anion form predominates is shown in Figure 4 S (in the Supporting Information). Unfortunately, the hydrolysis in the dark of BHQ-OAc to BHQ-OH in alkaline aqueous solution competes significantly with photolysis and would be observed during the ns-TR^3^ experiment. Inspection of Figure 4 S (in the Supporting Information) also suggests that the case in alkaline solution is fairly complex. Because the ns-TR^2^ spectrum of BHQ-OAc in alkaline solution was obtained at the very beginning of the experiment with fresh sample solutions, it is assumed that the main species at the moment of detection is still **A**(S_0_) of BHQ-OAc. Upon irradiation, **A**(S_0_) will undergo ISC process generating **A**(T_1_) or **T**(T_1_). The production of the latter species requires an extra excited protonation process, and the proton concentration in alkaline solution is limited; hence, it is reasonable to assume that the population of **T**(T_1_) at very early time is much lower, and the species detected in the ns-TR^2^ in alkaline aqueous solution is probably **A**(T_1_). Further support comes from the great similarity between the ns-TR^2^ spectrum with the calculated Raman spectrum of **A**(T_1_) (Figure 5 S in the Supporting Information).

**Figure 8:**
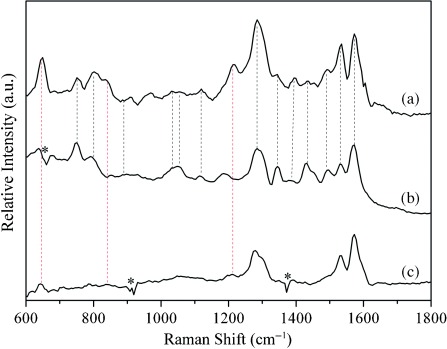
The ns-TR^2^ spectra of BHQ-OAc acquired in a) H_2_O/MeCN (3:2, *v*/*v*, pH 6–7) solution, b) NaOH-H_2_O/MeCN (3:2, *v*/*v*, pH 11–12) solution, and c) MeCN.

**Figure 9:**
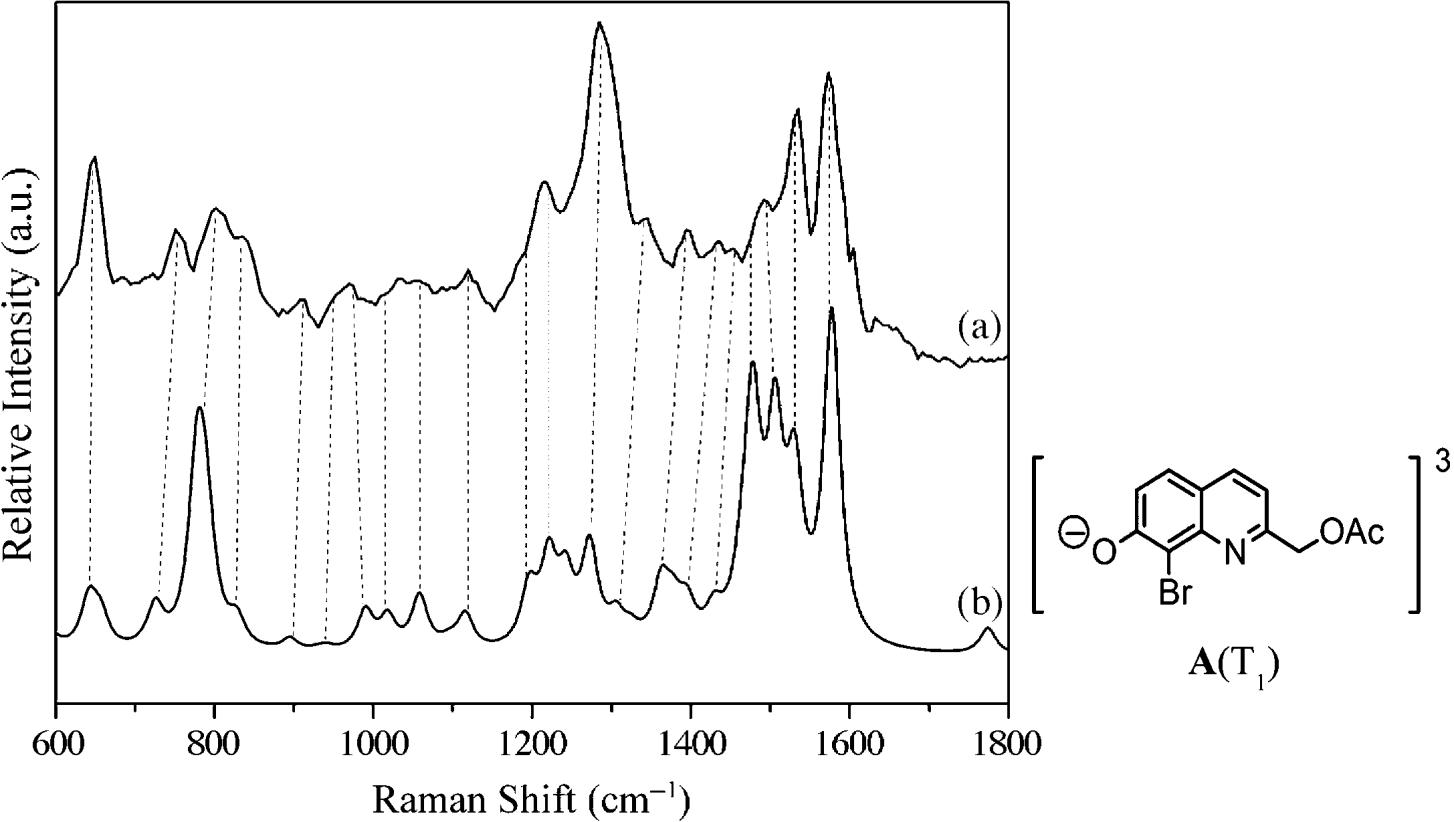
a) The ns-TR^2^ spectrum of BHQ-OAc obtained in mixed H_2_O/MeCN (3:2, *v*/*v*, pH 6–7) solution. b) The DFT calculated normal Raman spectrum of A(T_1_).

The ns-TR^2^ spectra acquired in alkaline and neutral aqueous solutions and MeCN (Figure 8) reveal that the spectrum (a) resembles (b) more closely while it has extra Raman bands. This suggests that spectrum (a) has contributions from several different forms of BHQ-OAc coexisting in the neutral aqueous solution and that the predominant transient species probed is probably the analogous transient species in the alkaline solution, **A**(T_1_). The residual peaks of (a) (labeled as a red line) that are missing in (b) can be found in spectrum (c), suggesting that some amount of **N**(T_1_) is also detected in the ns-TR^2^ spectrum acquired in neutral aqueous solutions. The ns-TR^2^ spectrum obtained in neutral aqueous solution was compared with the predicted DFT Raman spectrum of **A**(T_1_) species of BHQ-OAc (Figure 9) and the two exhibit good agreement with the vibrational frequency differences being smaller than 7 cm^−1^ on average. A comparison of the experimental and calculated vibrational frequencies, preliminary vibrational assignments, and qualitative descriptions of the vibrational modes in the 600 to 1800 cm^−1^ region (Table 1S in the Supporting Information) consistently indicate that the main species observed in ns-TR^2^ in neutral aqueous solution can be assigned to **A**(T_1_) of BHQ-OAc.

**Scheme 5:**
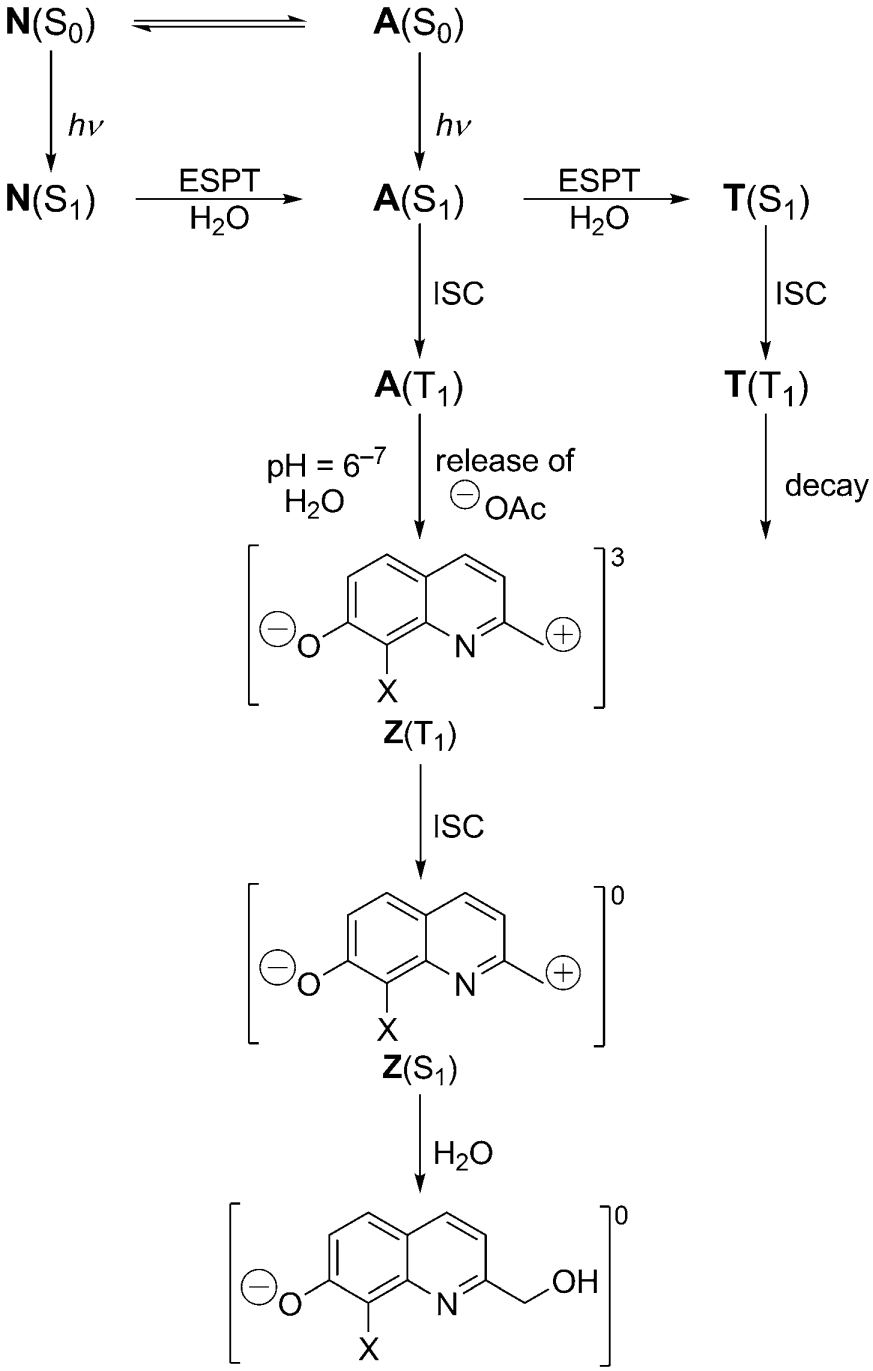
Proposed reaction routes of XHQ-OAc in neutral aqueous solution.

After the formation of **A**(S_1_), an ESPT reaction can proceed to produce some **T**(S_1_) in competition with the ISC process that forms **A**(T_1_). Since **T**(S_1_) was the other easily observed species in the ns-EM spectra of BHQ-OAc acquired in neutral aqueous solution, it is suggested that **T**(T_1_) may be also be created through an ISC process and observed in the ns-TR^3^ spectra. Since most of the reported ESPT reactions for the parent compound 7-HQ take place in the singlet excited state rather than triplet state, any **T**(T_1_) observed in the ns-TR^3^ spectra would probably be produced from the ISC process from **T**(S_1_), instead of being produced from **A**(T_1_) through an ESPT reaction. In addition to the **T**(T_1_) transient species, the species with Raman bands at 842 and 1626 cm^−1^ was observed. Combining with the ns-TA results that show the occurrence of the deprotection reaction in neutral aqueous solutions, the species detected here may correlate with a zwitterion-like BHQ intermediate generated from the deprotection reaction of BHQ-OAc in the physiologically relevant system.3b A comparison of the 500 ns experimental spectrum to the calculated normal Raman spectrum of the triplet zwitterion-like BHQ intermediate **Z**(T_1_) (Scheme 5) predicted from DFT calculations (Figure 6 S in the Supporting Information) reveals good agreement for the experimental vibrational frequencies, with the calculated frequencies being within about 9 cm^−1^ on average for the thirteen experimental Raman bands. This supports the assignment of the experimental spectrum at 500 ns being mainly due to the **Z**(T_1_) species. A comparison of the experimental and calculated vibrational frequencies with preliminary vibrational assignments and a qualitative description of the vibrational modes in the 600 to 1800 cm^−1^ region are given in Table 2S (in the Supporting Information). The TR^3^ spectra of BHQ-OAc in the neutral aqueous solution show that the triplet zwitterion-like BHQ intermediate **Z**(T_1_), which has a characteristic band at 1626 cm ^−1^, appears within 5 ns due to the deprotection reaction that will form this zwitterion species. Thus, the release of acetate is indicated to occur on approximately a 5 ns timescale. It is now reasonable to assign **A**(T_1_) as the major precursor of the photolysis reaction of BHQ-OAc in neutral water-containing solutions with the ESPT process to **T**(T_1_) as a competing process. The introduction of the heavy atom bromine into the compound BHQ-OAc not only facilitates the release of acetate, but also provides an opportunity for the ISC process following the ESPT, transferring **T**(S_1_) to **T**(T_1_).

The calculated Raman spectra of **T**(T_1_) and **Z**(T_1_) reproduce the large vibrational frequency up-shift of the nominal ring C—C stretch Raman bands observed in the ns-TR^3^ spectra compared with the ns-TR^2^ spectrum with **A**(T_1_) of BHQ-OAc as the predominant species (Table 2).

**Table 2:** Comparison of the experimental and calculated Raman bands of the nominal ring C—C stretch of A(T_1_), T(T_1_), and Z(T_1_).

Species	Experimental Raman shift [cm^−1^]	Calculated Raman shift [cm^−1^]
**A**(T_1_)	1576	1578
**T**(T_1_)	1600	1598
**Z**(T_1_)	1626	1620

**Figure 10:**
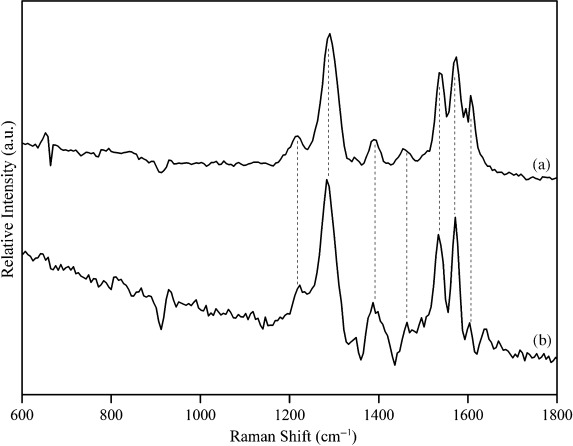
Comparison of the transient spectrum of a) CHQ-OAc obtained in alkaline H_2_O/MeCN (3:2, *v*/*v*, pH 11–12) and b) CHQ-OAc in neutral H_2_O/MeCN (3:2, *v*/*v*, pH 6–7) solutions.

The ns-TR^2^ and ns-TR^3^ spectra of CHQ-OAc are not complicated by the dehalogenation reaction that occurs with BHQ-OAc and ns-TR^3^ spectra for CHQ-OAc under alkaline conditions as that for BHQ-OAc (Figure 7 S in the Supporting Information). However, CHQ-OAc is rapidly hydrolyzed under these conditions to CHQ-OH and acetate, therefore, it is likely that the spectra obtained have significant contributions from CHQ-OH. Nevertheless, the transient spectrum obtained in neutral solution compared with that obtained in freshly prepared alkaline conditions (Figure 10) shows great similarity, demonstrating that the main transient species observed at neutral pH is **A**(T_1_) of CHQ-OAc. Two sets of Raman bands were detected in the TR^3^ spectra (Figure 7 b) with their most intense Raman band at 1606 and 1632 cm^−1^, respectively. Based on the analysis of BHQ-OAc, the two sets of bands might be due to the species **T**(T_1_) generated from the ESPT process and the zwitterion species **Z**(T_1_) that results from the deprotection reaction (Figure 8 S (inset) in the Supporting Information). To help make tentative assignments, the Raman spectra of **T**(T_1_) (Figure 9 S in the Supporting Information) and **Z**(T_1_) (Figure 8 Sb in the Supporting Information) were calculated and show intense signals at 1600 and 1630 cm^−1^, respectively. Therefore, the species that contributes at 1600 cm^−1^ was tentatively assigned to the **T**(T_1_) of CHQ-OAc species. The experimental Raman spectrum at 1000 ns delay time obtained in neutral aqueous solution was compared with the calculated Raman spectrum of **Z**(T_1_) of CHQ-OAc (Figure 8 S in the Supporting Information). There is a good similarity between the experimental and calculated Raman spectra, and this lends some support to the assignment of the transient species with Raman band at 1632 cm^−1^ to **Z**(T_1_) of CHQ-OAc. The ns-TR^3^ results reveal that **A**(T_1_) of CHQ-OAc acts as the precursor of the deprotection process, though it faces a side reaction from the ESPT process. The detection of **Z**(T_1_) of BHQ-OAc and CHQ-OAc only in neutral water-containing solution demonstrates that water and the neutral pH (or close to neutral) are important for the deprotection reaction to occur.

**DFT calculations**: DFT calculations were utilized to examine the activation barrier for the deprotection reaction from A(T_1_) of BHQ-OAc to the triplet zwitterion-like BHQ intermediate Z(T_1_) at the (U)B3LYP/6–311G** level of theory.^11^ The optimized geometries for the A(T_1_), transition state species, and the triplet zwitterion-like BHQ intermediate were readily found from the calculations. These data provide additional support for a two-step solvent-assisted heterolysis (S_N_1) mechanism for the deprotection reaction (see the Supporting Information for details). The calculation results revealed that the deprotection proceeds through a heterolytic cleavage from **A**(T_1_) of BHQ-OAc to form a triplet zwitterion-like BHQ intermediate, which undergoes ISC to its singlet and then proceeds via a singlet water–solvolysis reaction to produce the BHQ-OH side product.

## Conclusion

Combining the results from the time-resolved spectroscopy experiments (ns-EM, ns-TA, ns-TR^2^, and ns-TR^3^) with results from the photochemical quantum efficiency, product outcome, and quenching experiments and DFT calculations, a proposed reaction mechanism for the photophysical and photochemical reactions of BHQ-OAc after UV photolysis in aqueous environment has been deduced (Scheme 5). The results presented here indicate that **A**(T_1_) acts as the precursor for the desired deprotection reaction in neutral aqueous solution and has competition from an ESPT process that will form the **T**(T_1_) species. A favorable pathway with a low free energy barrier of approximately 4.6 kcal mol^−1^ (see the Supporting Information for details) was assigned to a solvent-assisted heterolysis from **A**(T_1_) to produce the triplet BHQ intermediate **Z**(T_1_). The completely different behavior of BHQ-OAc in MeCN and MeCN/H_2_O solvents demonstrates that water is vitally important for the desired photodeprotection reaction, which not only facilitates the excited state deprotonation reaction but also stabilizes the zwitterion transient species after the removal of the acetate leaving group. The occurrence of the photodeprotection reaction in the neutral aqueous solution suggests it can make meaningful and profound applications in a physiological environment. The poor suitability of CHQ-OAc as a PPG appears mainly due to its favored fluorescence and ESPT pathways and less efficient ISC capability to make the **A**(T_1_) precursor species for the deprotection reaction when compared with BHQ-OAc. This suggests that the introduction of a heavy atom is necessary for the desired photodeprotection reaction to take place with a high efficiency. A previous steady-state study on the comparison of BHQ-OAc with 7-HQ7b demonstrates that the involvement of the bromine atom inhibits the ground-state tautomerization reaction and lowers the population of **T**(S_0_), which also reduces unwanted side reactions of the deprotection reaction. On the other hand, the observed dehalogenation reaction for BHQ-OAc should be taken into consideration in future studies. This work sheds light on the design of promising two-photon PPGs based on the 7-hydroxyquinoline scaffold.

## Experimental Section

**Materials**: Dry acetonitrile was obtained by distillation from calcium hydride. Mixed solvents were prepared by adjusting the pH of 40 % MeCN/60 % H_2_O to pH 4 using 1 m HCl. Solutions for photolyses were prepared as follows: A 10 mm stock solution of BHQ-OAc or CHQ-OAc was prepared in dry MeCN, and dilutions to 100 μm with a final volume of 3 mL were made using each respective solvent. KMOPS buffer consisted of 100 mm KCl and 10 mm MOPS titrated to pH 7.2 with KOH.

**Synthesis**: BHQ-OAc was prepared by modification of the previously reported route3a using a MOM protecting group on the phenol instead of TBDPS. CHQ-OAc was synthesized and purified as described previously.3c HQ-OAc was prepared in one step by removal of the TBDPS group from a known compound.3a See the Supporting Information for details.

**Photochemistry**: Solutions of BHQ-OAc or CHQ-OAc were irradiated in a quartz test tube in a Rayonet photoreactor equipped with 254 nm bulbs. To monitor the progress of the photoreactions, aliquots (20 μL) were removed periodically and analyzed by HPLC using a 30:70 isocratic mix of MeCN and H_2_O containing 0.1 % TFA mobile phase, a 4.8 mm OD C-18 reverse phase column, and a diode array UV detector (observing at 254 nm). Analysis of the eluate from the HPLC column by mass spectrometry was performed one of two ways: 1) the HPLC system was directly interfaced in line with an ESI mass spectrometer, or 2) fractions corresponding to peaks on the chromatogram were collected and analyzed by ESI-MS offline. The quantum efficiency (*Q*_u_) under different solvent conditions was determined as previously described.3b, 9 Briefly, aliquots (20 μL) of the photolysis at 254 nm were removed periodically and analyzed by HPLC. The quantum efficiency was calculated using the equation *Q*_u_=(*Iσt*_90 %_)^−1^,^12^ in which *I* is the irradiation intensity in Einsteins cm^−2^ of the lamp measured by potassium ferrioxalate actinometry in the same setup, *σ* is the decadic extinction coefficient (1 000×ɛ) at 254 nm, and *t*_90 %_ is the time in seconds required for the conversion of 90 % of the starting material to product as measured by HPLC.

**Nanosecond transient emission (ns-EM) and nanosecond transient absorption (ns-TA) experiments**: The ns-EM and ns-TA measurements were performed on an LP-920 Laser flash photolysis setup (Edinburgh Instruments). The 266 nm pump laser pulse was obtained from the fourth harmonic output of an Nd:YAG Q-switched laser, and the probe light was provided by a 450 W xenon lamp. The sample was excited by the pump laser, and the probe light from the xenon lamp was passed through the sample at right angles to the path of the exciting pulse. The two beams were focused onto a 1 cm quartz cell. After passing through the sample the analyzing light was directed to a monochromator/spectrograph. The transmitted probe light was then measured either by a single detector (for kinetic analysis at a single wavelength) or by an array detector (for spectral analysis at a given time). The transmission properties of the sample before, during, and after the exciting pulse were converted by the detector into electrical signals, which were measured by an oscilloscope (in the case of the single detector) or acquired by a CCD camera (in the case of an array detector). The changes in the transmission properties were converted into changes of optical density. The signals analyzed by a symmetrical Czerny-Turner monochromator were detected by a Hamamatsu R928 photomultiplier, and the signal processed via an interfaced PC and analytical software. Unless otherwise indicated, the ns-EM and ns-TA experiments were conducted in air saturated solutions and the sample solutions were prepared to yield the same absorbance at the specified *λ*_ex_. In that way, the same number of photons is absorbed for the same irradiating conditions in each case.^13^ For the oxygen quenching ns-TA experiment, the sample solution was placed in a 1 cm quartz cell where oxygen was bubbled constantly for 30 min through the sample in that cell, and then the cell was sealed to prepare it for the ns-TA experiment.

**Nanosecond transient resonance Raman (ns-TR^2^) and time-resolved resonance Raman (ns-TR^3^) experiments**: Resonance Raman experiments, ns-TR^2^ and ns-TR^3^, were performed using our previously described experimental apparatus and method.^14^ Briefly, the fourth harmonic of a Nd:YAG nanosecond-pulsed laser supplied the 266 nm pump beam and the third anti-Stokes hydrogen Raman-shifted laser line from the second harmonic of a second Nd:YAG laser supplied the 319.9 nm probe beam used in the ns-TR^3^ experiments. The ns-TR^2^ spectrum was obtained from the difference of the spectra under 266 nm high power and low power of the pump laser by taking an appropriately scaled difference spectrum to subtract the precursor and solvent Raman bands. The 266 nm pump pulse excited the sample to initiate the photochemical reactions, and the 319.9 nm probe pulse interrogated the sample and the intermediate species produced by the pump pulse. The laser beams were lightly focused and aligned so that they were overlapped onto a flowing liquid stream of sample. The diameter of the pump beam was adjusted to be slightly larger than that of the probe beam at the overlapping volume in the liquid jet to minimize the ground-state normal Raman signal. A pulse delay generator was employed to electronically control the time delay between the pump and probe laser beams from the two different Nd:YAG lasers operated at a repetition rate of 10 Hz. The Raman scattered light was acquired using a backscattering geometry, then detected by a liquid-nitrogen-cooled charge-coupled device (CCD) detector. The TR^3^ signal was acquired for 10 s by the CCD before being read out to an interfaced PC computer. Ten scans of the signal were accumulated to produce a resonance Raman spectrum. The ns-TR^3^ spectra were obtained from subtraction of an appropriately scaled probe-before-pump spectrum from the corresponding pump-probe resonance Raman spectrum to remove non-transient bands. The Raman bands of acetonitrile were employed to calibrate the Raman shifts of the ns-TR^3^ spectra with an estimated accuracy of 5 cm^−1^. A Lorentzian function was used to integrate the Raman bands of the species of interest in the ns-TR^3^ spectra to determine their areas and elucidate the growth and decay time constants of the species observed in the experiments.

**Density functional theory calculations**: DFT calculations employing (U)B3LYP method with a 6–311G** basis set were done to determine the optimized geometries and vibrational wavenumbers for all of the likely species that may be potential intermediates associated with the photochemical reaction of BHQ-OAc and CHQ-OAc. No imaginary frequencies were observed at any of the optimized structures studied. A Lorentzian function with a 15 cm^−1^ bandwidth was used for the Raman vibrational frequencies and the relative intensities to obtain the computational Raman spectra that were compared to the experimental TR^3^ spectra. A frequency scaling factor of 0.974 was used in the comparison of the calculated results with the experimental spectra. All the calculations presented in this paper were done using the Gaussian 03^11^ program suite installed on the High Performance Computing cluster in the Computer Centre at The University of Hong Kong.

## Notes

Detailed facts of importance to specialist readers are published as ”Supporting Information”. Such documents are peer-reviewed, but not copy-edited or typeset. They are made available as submitted by the authors.
