# Supplementary material for: Unraveling the Mechanism of the Photodeprotection Reaction of 8-Bromo- and 8-Chloro-7-hydroxyquinoline Caged Acetates
Source: Chemistry. 2012 Apr 18;18(22):6854–65. doi: 10.1002/chem.201200366 (PMC3531613; doi:10.1002/chem.201200366)
Supplement: Supplementary file 1 [file chem0018-6854-SD1.pdf]

CHEMISTRY
A EUROPEAN JOURNAL

Supporting Information

© Copyright Wiley-VCH Verlag GmbH & Co. KGaA, 69451 Weinheim, 2012

Unraveling the Mechanism of the Photodeprotection Reaction of 8-Bromo- and 8-Chloro-7-hydroxyquinoline Caged Acetates

Jiani Ma,[a] Adam C. Rea,[b] Huiying An,[a] Chensheng Ma,[a] Xiangguo Guan,[a] Ming-De Li,[a] Tao Su,[a] Chi Shun Yeung,[a] Kyle T. Harris,[b] Yue Zhu,[b] Jameil L. Nganga,[b] Olesya D. Fedoryak,[b] Timothy M. Dore,*[b] and David Lee Phillips*[a]

chem_201200366_sm_miscellaneous_information.pdf

Table of Contents

Supplementary Figures and Tables.. 4

Figure 1S... 4
The ns-EM spectra of BHQ-OAc acquired in MeCN.

Figure 2S... 4
The time evolution with an initial growth followed by a decay of the ΔA monitored at 520 nm from the transient absorption spectra of BHQ-OAc in oxygen saturated MeCN following 266-nm laser irradiation.

Figure 3S... 4
Transient absorption spectra of BHQ–OH in H_2O/MeCN (3:2, v/v, pH 6–7) under 266-nm laser irradiation.

Figure 4S... 5
Comparison of the ns-TR^2 spectrum to the ns-TR^3 spectra of BHQ-OAc in H_2O/MeCN (3:2, v/v, pH 11–12) solution with varying time delays indicated next to the spectra with the 266-nm pump and 355-nm probe laser pulses.

Figure 5S... 5
(a) The ns-TR^2 spectrum of BHQ-OAc obtained in mixed NaOH-H_2O/MeCN (3:2, v/v, pH 11–12) solution and (b) the DFT calculated normal Raman spectrum of **A**(T_1).

Figure 6S... 6
(a) The 500 ns TR^3 spectrum of BHQ-OAc in a mixed H_2O/MeCN (3:2, v/v, pH 6–7) solution, and (b) the DFT calculated normal Raman spectrum of the triplet zwitterion-like BHQ intermediate.

Figure 7S. 6
The ns-TR^2 spectrum (labelled "transient") and ns-TR^3 spectra of CHQ-OAc in H_2O/MeCN (3:2, v/v, pH 11–12) solution with varying time delays indicated next to the spectra with the 266-nm pump and 355-nm probe laser pulses.

Figure 8S. 7
Comparison of (a) the ns-TR^3 spectrum of CHQ-OAc obtained in neutral H_2O/MeCN (3:2, v/v, pH 6–7) solution at 1000 ns with (b) the calculated Raman spectrum of **Z**(T_1) (inset).

Figure 9S. 7
The calculated Raman spectrum of **T**(T_1) of CHQ-OAc with the structure shown in top of the figure.

Table 1S. 8
Comparison of the experimental 10-ns TR^3 spectrum vibrational frequencies in alkaline solution and the DFT calculated vibrational frequencies for **A**(T_1) of BHQ-OAc with preliminary vibrational assignments and qualitative description of the vibrational modes in the 600 to 1800 cm^{-1} region.

Table 2S. 8
Comparison of the experimental 500-ns TR^3 spectrum vibrational frequencies in neutral aqueous solution and the DFT calculated vibrational frequencies for the triplet zwitterion-like BHQ intermediate with preliminary vibrational assignments and qualitative description of the vibrational modes in the 600 to 1800 cm^{-1} region.

DFT Calculations for the Photodeprotection and Solvolysis Processes. 9

Figure 10S. 9
Optimized geometries of **A**(T_1) of BHQ-OAc (**RC**), **TS**, and triplet zwitterion-like BHQ intermediate (**IM**) obtained from the DFT (U)B3LYP/6–311G** computations for the heterolytic cleavage step: BHQ–OAc ? BHQ$^+$ + $^-$OAc.

Scheme 1S. 10
Two possible pathways for the water-solvolysis step.

Figure 11S 10
The optimized geometries of the BHQ intermediate complexes, transition states and the final product BHQ-OH obtained from the DFT (U)B3LYP/6–311G** computations for the singlet (RX1) and triplet (RX2) water solvolysis step: BHQ + H_2O ? BHQ-OH.

Table 3S 10
Free energy difference at 298 K of the singlet and triplet BHQ intermediate (**IM**), reactant complex (**IM**+H_2O), and final product BHQ-OH in vacuum, MeCN, and water.

Table 4S. 11
Activation free energies at 298K of the singlet and triplet solvolysis processes (RX1 and RX2) in vacuum, MeCN, and water.

Figure 12S. 11
Relative energy profiles (in kcal/mol) obtained from (U)B3LYP/6–311G** calculations to study the solvolysis step: BHQ + H_2O ? BHQ-OH, with associated reaction pathways shown as **a** and **b** in Scheme 1S: (a) before solvent effect correction (b) after solvent correction.

Tables 5S-11S 12
The Cartesian coordinates, total energies, and vibrational zero-point energies for the optimized geometry from the (U)B3LYP/6-311G** calculations for the species of interest.

Table 5S. 12
T(T_1) of BHQ-OAc

Table 6S. 13
A(T_1) of BHQ-OAc

Table 7S. 14
Z(T_1) of BHQ-OAc

Table 8S. 15
Singlet BHQ-OH Anion

Table 9S. 16
A(T_1) of CHQ-OAc

Table 10S. 17
T(T_1) of CHQ-OAc

Table 11S. 18
Z(T_1) of CHQ-OAc

Synthetic Procedures. 19

References. 20

Supplementary Figures and Tables

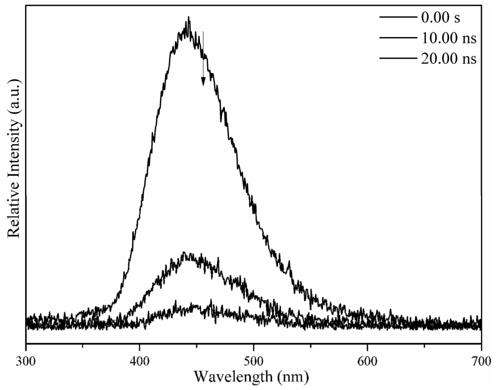

Figure 1S. The ns-EM spectra of BHQ-OAc acquired in MeCN.

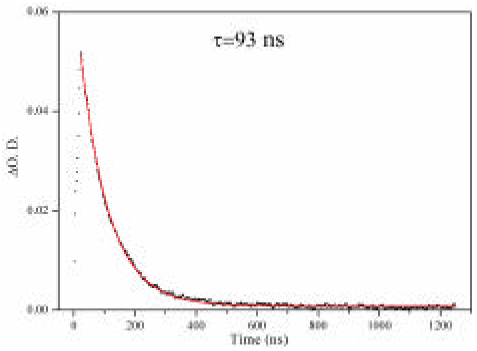

Figure 2S. The time evolution with an initial growth followed by a decay of the ΔA monitored at 520 nm from the transient absorption spectra of BHQ-OAc in oxygen saturated MeCN following 266-nm laser irradiation.

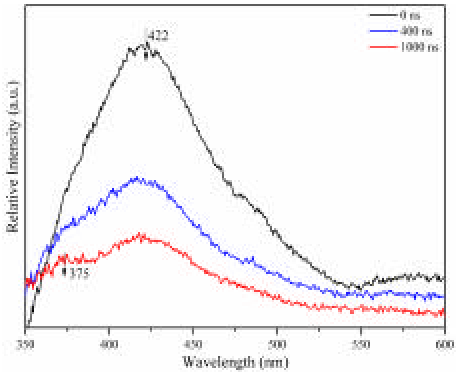

Figure 3S. Transient absorption spectra of BHQ–OH in H_2O/MeCN (3:2, v/v, pH 6–7) under 266-nm laser irradiation.

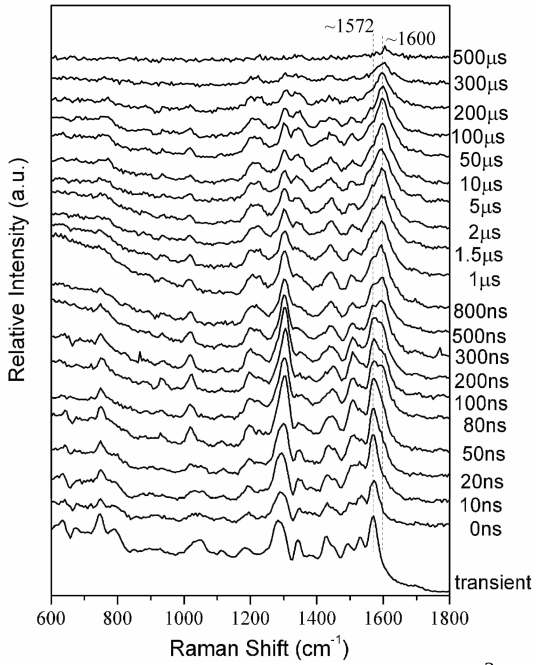

Figure 4S. Comparison of the ns-TR[2] spectrum to the ns-TR[3] spectra of BHQ-OAc in H_2O/MeCN (3:2, v/v, pH 11–12) solution with varying time delays indicated next to the spectra with the 266-nm pump and 355-nm probe laser pulses.

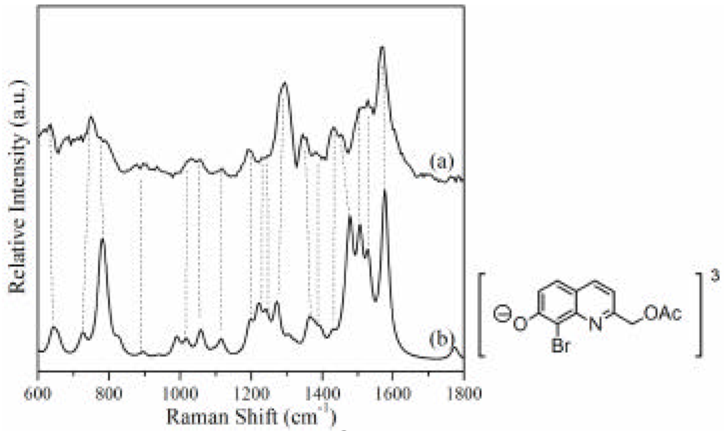

Figure. 5S (a) The ns-TR[2] spectrum of BHQ-OAc obtained in mixed NaOH-H_2O/MeCN (3:2, v/v, pH 11–12) solution and (b) the DFT calculated normal Raman spectrum of **A**(T_1).

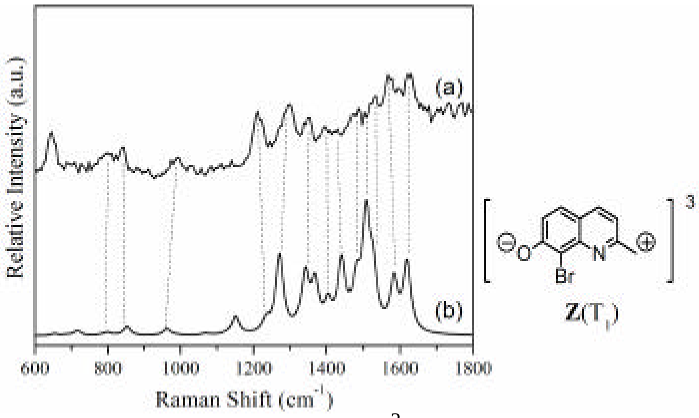

Figure 6S. (a) The 500 ns TR[3] spectrum of BHQ-OAc in a mixed H_2O/MeCN (3:2, v/v, pH 6–7) solution, and (b) the DFT calculated normal Raman spectrum of the triplet zwitterion-like BHQ intermediate **Z**(T_1).

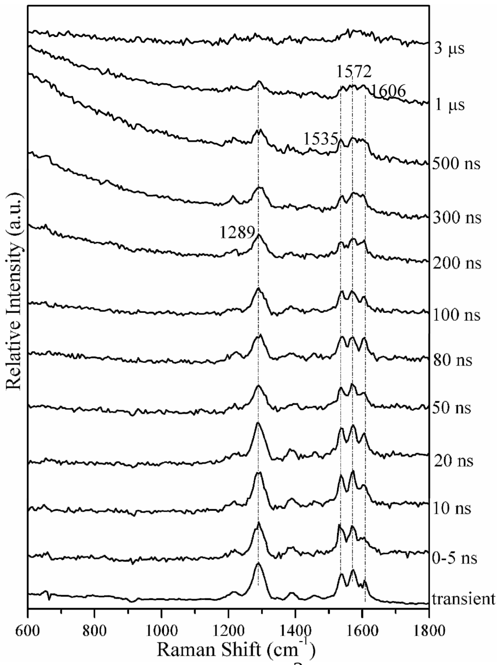

Figure 7S. The ns-TR[2] spectrum (labeled "transient") and ns-TR[3] spectra of CHQ-OAc in H_2O/MeCN (3:2, v/v, pH 11–12) solution with varying time delays indicated next to the spectra with the 266-nm pump and 355-nm probe laser pulses.

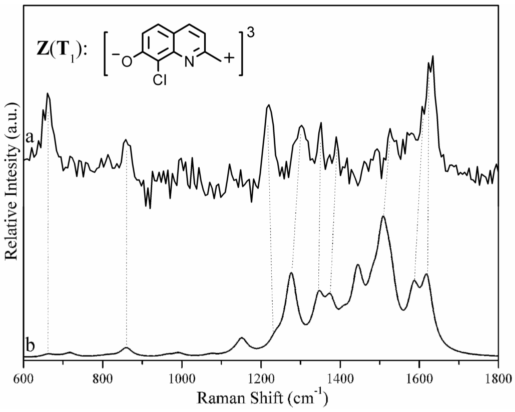

Figure 8S. Comparison of (a) the ns-TR^3 spectrum of CHQ-OAc obtained in neutral H_2O/MeCN (3:2, v/v, pH 6–7) solution at 1000 ns with (b) the calculated Raman spectrum of **Z**(T_1) (inset).

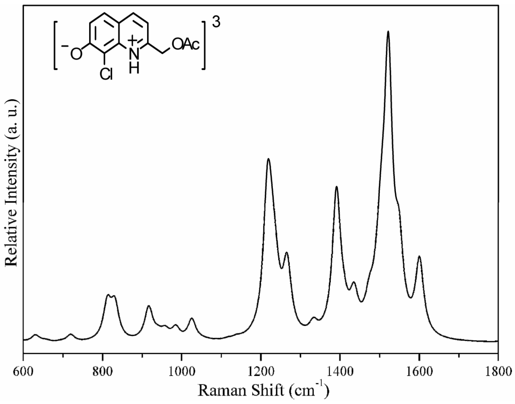

Figure 9S. The calculated Raman spectrum of **T**(T_1) of CHQ-OAc with the structure shown in top of the figure.

Table 1S. Comparison of the experimental 10-ns TR^3 spectrum vibrational frequencies in alkaline solution and the DFT calculated vibrational frequencies for **A**(T_1) of BHQ-OAc with preliminary vibrational assignments and qualitative description of the vibrational modes in the 600 to 1800 cm^{-1} region.

Experimental Frequency (cm^{-1})	Calculated	
	Frequency (cm^{-1})	Assignment description
635	644 (?$_{25}$)	C–C and C–N stretching
749	726 (?$_{28}$)	Deformation
777	778 (?$_{30}$)	CH_2–O stretching and C–H(ring) bending
895	895 (?$_{35}$)	C–CH_3, C–O–C stretching and C–H(CH_2) bending
1018	1018 (?$_{39}$)	C–H(CH_3) bending
1057	1058 (?$_{40}$)	C–C(CH_3) and C–H(CH_3) bending
1118	1116 (?$_{43}$)	C–C stretching and C–H(ring) scissor
1196	1196 (?$_{44}$)	C–C stretching and C–H bending
1228	1221 (?$_{45}$)	C–C(ring), C(ring)–C(CH_2) stretching and C–H bending
1245	1242 (?$_{46}$)	C–C(CH_3) stretching and C–H bending
1295	1271 (?$_{47}$)	C–C(ring), O–C(=O) stretching and C–H bending
1350	1362 (?$_{51}$)	C–N stretching and C–H bending
1382	1378 (?$_{53}$)	C–N, C–C(ring) stretching and C–H(CH_2, CH_3) bending
1431	1429 (?$_{55}$)	O^-–C and C–N, C–C (ring) stretching and C–H(ring) bending
1453	1478 (?$_{59}$)	O^-–C(ring), C–C(ring) stretching and C–H(CH_3) scissor
1507	1506 (?$_{60}$)	C–C(ring) stretching and C–H bending
1529	1531 (?$_{61}$)	O^-–C(ring) and C–C(ring) stretching
1572	1578 (?$_{62}$)	C–C(ring) stretching

Table 2S. Comparison of the experimental 500-ns TR^3 spectrum vibrational frequencies in neutral aqueous solution and the DFT calculated vibrational frequencies for the triplet zwitterion-like BHQ intermediate with preliminary vibrational assignments and qualitative description of the vibrational modes in the 600 to 1800 cm^{-1} region.

Experimental Frequency (cm^{-1})	Calculated	
	Frequency (cm^{-1})	Assignment description
800	800 (?$_{21}$)	C–C(ring) and C–Br stretching
844	853 (?$_{24}$)	C–C(ring) stretching
973	963 (?$_{27}$)	C–Br stretching and C–H bending
1212	1235 (?$_{33}$)	C–C(ring) stretching and C–H bending
1300	1273 (?$_{35}$)	C(CH2)–C(ring) stretching and C–H bending
1341	1343 (?$_{37}$)	C–N stretching and C–H bending
1399	1405 (?$_{39}$)	C–C(ring) stretching and C–H bending
1431	1442 (?$_{40}$)	C–C(ring) stretching and C–H bending
1475	1481 (?$_{41}$)	C–H(CH2) scissor
1502	1507 (?$_{42}$)	C–C stretching and C–H bending
1529	1526 (?$_{43}$)	O–C,C–C stretching and C–H bending, C–H(CH2) scissor
1572	1583 (?$_{44}$)	O–C and C–C stretching
1626	1620 (?$_{45}$)	C–C stretching

DFT Calculations for the Photodeprotection and Solvolysis Processes

DFT calculations were utilized to examine the activation barrier for the deprotection reaction from A(T_1) of BHQ-OAc to the triplet zwitterion-like BHQ intermediate Z(T_1) at the (U)B3LYP/6–311G** level of theory.[1] The optimized geometries for the A(T_1) (**RC**), transition state species (**TS**) and the triplet zwitterion-like BHQ intermediate (**IM**) were readily found from the calculations (Figure 10S). As the reaction proceeds from **RC** to **TS**, the distance between the C13 and O14 varies substantially from 1.505 to 1.865 Å, and the bond length of C5–C13 decreases from 1.483 to 1.419 Å, and as the reaction proceeds from **TS** to **IM**, the C13–O14 bond completes its cleavage and the bond length of C5–C13 changes to 1.398 Å. During this reaction, the geometry of the CH_2 moiety changes from a pyramidal structure (**RC**) to a planar one (**IM**), which reveals that the hybridization of the atom C13 goes from sp^3 to sp^2. The triplet reaction process can also be followed by the changes observed in the spin densities. For **RC**, the spin density delocalizes and is 0.01 for the C13 atom and 0.19 for the C5 atom, and when the reaction goes to **TS**, the spin density becomes localized and is 0.19 for the C13 atom and 0.12 for the O14 atom. When the reaction proceeds further to the intermediate complexes (**IM** + $^-$OAc), the spin density concentrates mainly on the C13 atom and is 0.73 on this atom. The DFT calculated activation barrier of the deprotection reaction is predicted after the correction of the solvent effect on the optimizations done for the gas-phase stationary points (the dielectric constant used for water is 78.39) and the calculated result (? $G^{\ddagger}(H_2O)$) is approximately 4.6 kcal/mol. The estimated timescale of the deprotection reaction determined by employing a combination of the Eyring equation and the Arrhenius equation gives the barrier of the deprotection as 6.2–6.5 kcal/mol from the experimental results (6–10 ns). As the DFT calculation tends to underestimate the reaction barrier to some extent, the calculated free energy (? $G^{\ddagger}(H_2O)$=4.6 kcal/mol) is in good agreement with the experimental results. In addition, the low activation barrier further supports that the deprotection proceeds via a heterolytic cleavage from **A**(T_1) of BHQ-OAc to form a triplet zwitterion-like BHQ intermediate.

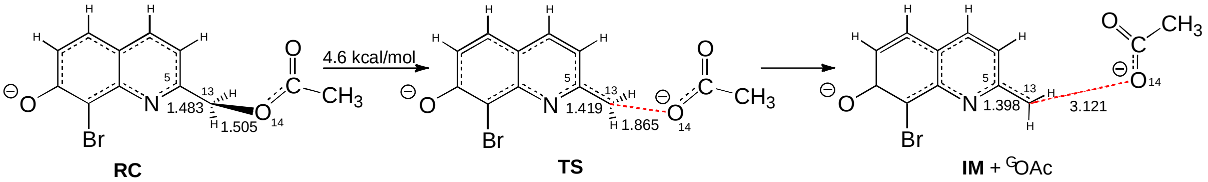

RC **TS** **IM** + $^-$OAc

Figure 10S. Optimized geometries of **A**(T_1) of BHQ-OAc (**RC**), **TS**, and triplet zwitterion-like BHQ intermediate (**IM**) obtained from the DFT (U)B3LYP/6–311G** computations for the heterolytic cleavage step: BHQ-OAc ? BHQ$^+$ + $^-$OAc.

After release of acetate, intermediate (**IM**) undergoes further reaction to produce the final product (a singlet anionic form of BHQ-OH) in aqueous solution.[2] There are two possible pathways for this water-solvolysis step (**a** and **b** in Scheme 1S). In pathway **a**, **Z**(T_1) undergoes ISC first to form a singlet transient **Z**(S_1), which then reacts with water to produce the final product. Conversely, reaction of **Z**(T_1) with water first in pathway **b** produces a triplet BHQ-OH, which undergoes ISC to form the final product. DFT calculations with the optimized geometries for the BHQ intermediate complex (**RC1** and **RC2**), transition state (**TS1** and **TS2**), and the final by-product BHQ-OH (**BHQ-OH(S_0)** and **BHQ-OH(T_1)**) (Figure 11S) were done to distinguish the two water solvolysis pathways described in Scheme 1S. RX1 depicts a singlet solvolysis process subsequent to an ISC (pathway **a**), and RX2 depicts a triplet solvolysis process followed by an ISC (pathway **b**).

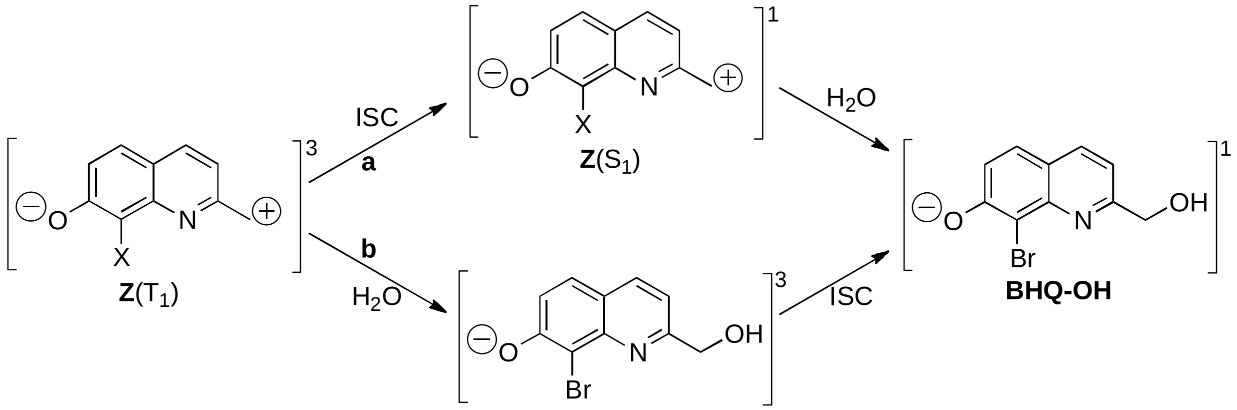

Scheme 1S. Two possible pathways for the water-solvolysis step.

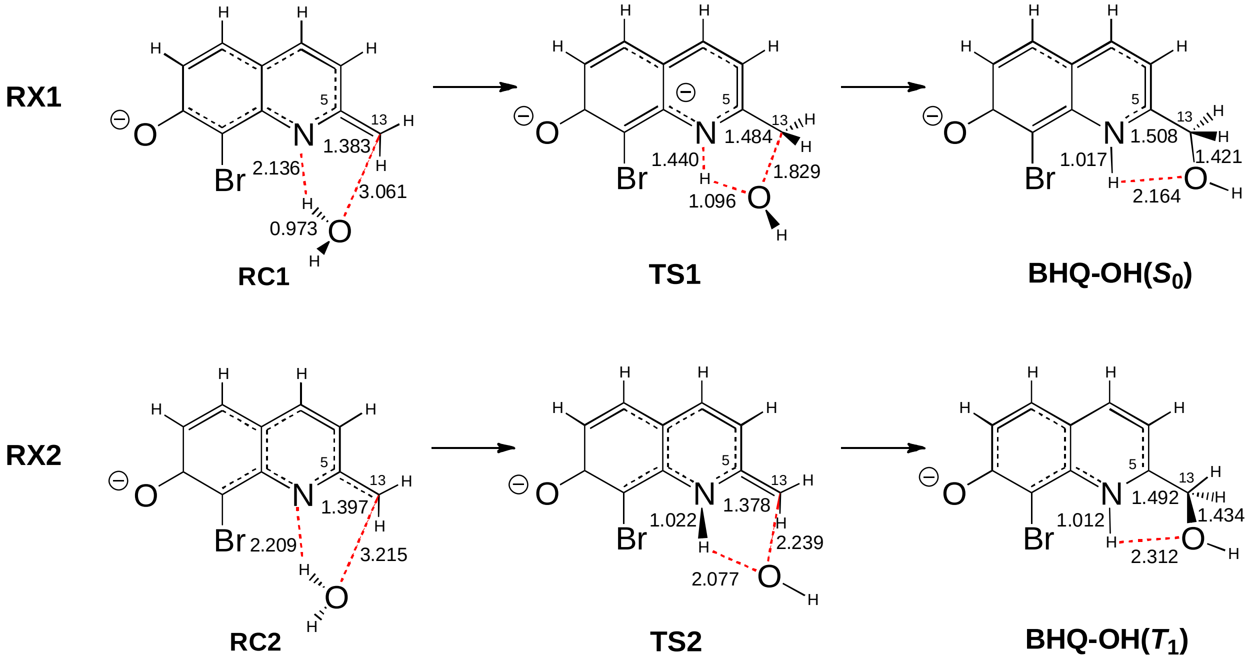

Figure 11S. The optimized geometries of the BHQ intermediate complexes, transition states and the final product BHQ-OH obtained from the DFT (U)B3LYP/6–311G** computations for the singlet (RX1) and triplet (RX2) water solvolysis step: BHQ + H_2O ? BHQ-OH.

Table 3S. Free energy difference at 298 K of the singlet and triplet BHQ intermediate (**IM**), reactant complex (**IM**+H_2O), and final product BHQ-OH in vacuum, MeCN, and water.

	**? G**‡ (Singlet- Triplet) (kcal/mol)		
	Vacuum	Solvent: MeCN (*e* = 36.64)	Solvent: H_2O (*e* = 78.39)
**IM**	18.2		
**IM**-water complex	15.6	10.8	10.3
BHQ–OH	- 36.1	- 44.3	- 47.8

The calculated free energy of the singlet state of the BHQ intermediate is ~18.2 kcal/mol higher than that of its triplet state although both optimized structures are found to be similar (Table 3S). This suggests that the lowest singlet state of the BHQ intermediate is an active one, and the result may be obtained because the large conjugated hydroxyquinoline system of the transient species can make the triplet more stable. This also provides further support for

the heterolytic cleavage step of the deprotection reaction occuring on the excited triplet potential surface. Investigation of the reactant complex formed by the BHQ intermediate and one water molecule reveals that although the calculated free energy of the singlet state is still higher than that of the triplet, the difference decreases to 15.6 kcal/mol, and then further shrinks to ~10 kcal/mol after correction for solvent effects. Therefore, the smaller difference between their free energies and the similarity of the structures may suggest that the ISC of the BHQ intermediate complex from its triplet state to the active singlet state is relatively easy to occur in solutions. The calculated free energy of the lowest singlet state of BHQ-OH is 36.1 kcal/mol lower than the triplet state, indicating that the ground state of the stable final byproduct is a singlet state and the formation of BHQ-OH is thermodynamically favorable.

Table. 4S Activation free energies at 298K of the singlet and triplet solvolysis processes (RX1 and RX2) in vacuum, MeCN, and water.

	**? G**‡(Vacuum)	**? G**‡(MeCN) *e* = 36.64	**? G**‡(H_2O) *e* = 78.39
Singlet Process (RX1)	3.9	- 13.4	- 27.9
Triplet Process (RX2)	37.1	35.8	37.9

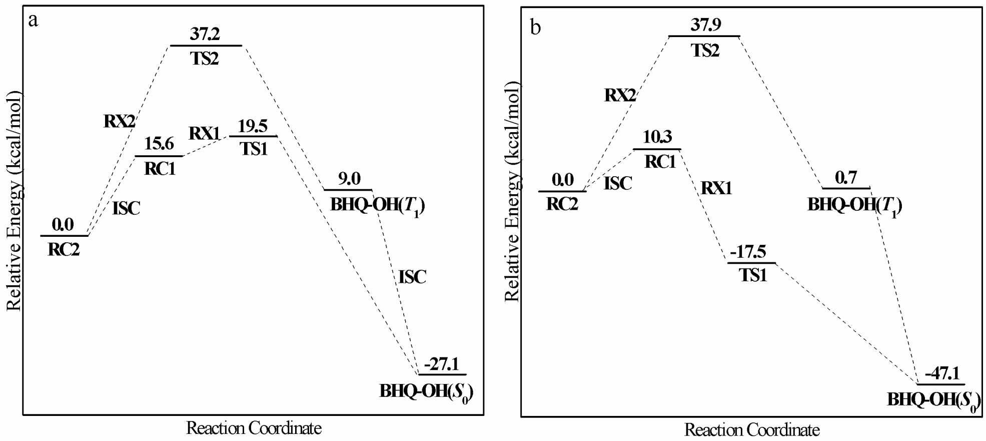

Figure 12S. Relative energy profiles (in kcal/mol) obtained from (U)B3LYP/6–311G** calculations to study the solvolysis step: BHQ + H_2O ? BHQ-OH, with associated reaction pathways shown as **a** and **b** in Scheme 1S: (a) before solvent effect correction (b) after solvent correction.

The reaction barriers of the singlet and triplet water-solvolysis processes (RX1 and RX2 in Figure 11S) and the relative energy profiles before and after water solvent corrections (Table 4S and Figure 12S) indicate that both before and after the correction of solvent effects, the triplet process RX2 has very high free energy barrier (37–38 kcal/mol), and the solvent corrections seem to have only a small influence. Therefore, the triplet reaction is probably endothermic and unfavorable both kinetically and thermodynamically. However, if the triplet BHQ intermediate undergoes ISC to the active singlet state first, the subsequent singlet solvolysis RX1 reaction has a very low activation free energy barrier (~3.9 kcal/mol). Especially after water solvent correction, the activation barrier substantially decreases to a negative value (- 27.9 kcal/mol), which suggests that water solvolysis of the singlet BHQ intermediate occurs spontaneously in aqueous solutions. Therefore, the decisive step of the singlet solvolysis in aqueous solutions is the ISC process, not the reaction between the BHQ intermediate and water molecule. Similar observations that the singlet-triplet ISC can play a

crucial role for solvolysis reactions have been reported in the literature.[3] The calculation results are consistent with the assumption that the triplet BHQ intermediate, similar to the photochemistry of benzoin diethylphosphate,[4] undergoes ISC to its singlet and then proceeds via a singlet water-solvolysis reaction to produce the BHQ-OH side-product.

Tables 5S-11S. The Cartesian coordinates, total energies, and vibrational zero-point energies for the optimized geometry from the (U)B3LYP/6-311G** calculations for the species of interest.

Table 5S. **T**(T_1) of BHQ-OAc
Total energy: E (UB+HF–LYP) = –3318.003713 au
Zero–point vibrational energy = 514972.7 (J/mol)
Zero–point correction = 0.196143 (Hartree/Particle)
No imaginary frequency

Center Number	Atomic	Coordinates (Angstroms)		
		X	Y	Z
1	C	–0.388185	3.075591	0.095051
2	C	–1.397614	2.031091	0.124167
3	C	–0.962902	0.686967	–0.061772
4	N	0.374137	0.438892	–0.286693
5	C	1.343794	1.429440	–0.315913
6	C	0.933791	2.749229	–0.115544
7	C	–2.769210	2.286961	0.337383
8	C	–3.707143	1.282364	0.380099
9	C	–3.334811	–0.097482	0.209089
10	C	–1.902510	–0.347251	–0.017029
11	Br	–1.362712	–2.146479	–0.248769
12	O	–4.154818	–1.035888	0.245875
13	C	2.728608	1.005026	–0.636747
14	O	3.119835	–0.035444	0.309985
15	C	4.395319	–0.485994	0.190273
16	O	5.172582	–0.051670	–0.619315
17	C	4.677334	–1.574776	1.193903
18	H	–0.692220	4.102242	0.249419
19	H	1.690555	3.524856	–0.136633
20	H	–3.075931	3.318247	0.474080
21	H	–4.758164	1.481406	0.545073
22	H	3.418790	1.845788	–0.568311
23	H	2.816107	0.578264	–1.642884
24	H	4.433753	–1.230525	2.200826
25	H	4.048959	–2.443629	0.983519
26	H	5.726220	–1.856956	1.134378
27	H	0.668290	–0.528135	–0.315809

Table 6S. **A**(T_1) of BHQ-OAc
Total energy: E (UB+HF–LYP) = –3317.4635023 au
Zero–point vibrational energy = 478926.2 (J/mol)
Zero–point correction = 0.182413 (Hartree/Particle)
No imaginary frequency

Center Number	Atomic	Coordinates (Angstroms)		
		X	Y	Z
1	C	0.584210	2.584888	–0.300510
2	C	–0.685070	1.981751	–0.040222
3	C	–0.749852	0.541221	–0.203709
4	N	0.294939	–0.214105	–0.571222
5	C	1.496635	0.402657	–0.808675
6	C	1.654275	1.785078	–0.678121
7	C	–1.832563	2.706927	0.349175
8	C	–3.060646	2.108508	0.592523
9	C	–3.237634	0.695535	0.459037
10	C	–2.021805	–0.058823	0.048568
11	Br	–2.214926	–1.938880	–0.144805
12	O	–4.322600	0.108838	0.664785
13	C	2.622799	–0.480558	–1.196357
14	O	3.355814	–1.049882	–0.011217
15	C	4.441867	–0.407217	0.412806
16	O	4.956186	0.550915	–0.123427
17	C	4.961284	–1.026934	1.696665
18	H	5.966455	–0.659471	1.899925
19	H	4.953418	–2.116085	1.630305
20	H	4.297559	–0.742589	2.517941
21	H	3.372150	0.029063	–1.799844
22	H	2.250197	–1.376545	–1.689611
23	H	2.630092	2.223767	–0.866132
24	H	0.695778	3.659710	–0.202348
25	H	–1.732485	3.784339	0.457425
26	H	–3.931546	2.680185	0.890851

Table 7S. **Z**(T_1) of BHQ-OAc

Total energy: E (UB+HF–LYP) = –3088.8458132 au
Zero–point vibrational energy = 342519.9 (J/mol)
Zero–point correction = 0.130459 (Hartree/Particle)
No imaginary frequency

Center Number	Atomic Number	Coordinates (Angstroms)		
		X	Y	Z
1	C	–2.630453	1.457168	0.000082
2	C	–1.219317	1.457565	–0.000126
3	C	–0.561077	0.174633	–0.000214
4	N	–1.232199	–0.986486	–0.000162
5	C	–2.590559	–0.969148	0.000152
6	C	–3.316348	0.271842	0.000207
7	C	–0.454138	2.664672	–0.000225
8	C	0.901508	2.648386	–0.000124
9	C	1.661508	1.398114	0.000074
10	C	0.865556	0.162121	–0.000097
11	Br	1.802217	–1.459238	–0.000040
12	O	2.895717	1.392599	0.000460
13	C	–3.249829	–2.200608	0.000068
14	H	–3.159819	2.404109	0.000091
15	H	–4.399694	0.252580	0.000508
16	H	–0.990698	3.608217	–0.000379
17	H	1.491755	3.556428	–0.000114
18	H	–4.330500	–2.258737	0.000084
19	H	–2.670090	–3.113133	–0.000139

Table 8S. Singlet BHQ-OH Anion

Total energy: E (UB+HF–LYP) = –3164.8263575 au
Zero–point vibrational energy = 389469.6 (J/mol)
Zero–point correction = 0.148341 (Hartree/Particle)
No imaginary frequency

Center Number	Atom	Coordinates (Angstroms)		
		X	Y	Z
1	C	1.859545	2.129973	–0.086965
2	C	0.500566	1.808506	–0.037291
3	C	0.129270	0.406054	–0.048118
4	N	1.101876	–0.559320	–0.094272
5	C	2.370258	–0.201359	–0.147380
6	C	2.821992	1.135418	–0.143593
7	C	–0.531735	2.788823	0.023797
8	C	–1.840845	2.427369	0.069929
9	C	–2.307927	1.033233	0.063162
10	C	–1.231919	0.075039	0.001636
11	O	–3.523608	0.768841	0.108586
12	Br	–1.754251	–1.781817	–0.008720
13	C	3.366784	–1.343992	–0.225683
14	O	4.623982	–1.060514	0.402113
15	H	2.148870	3.178871	–0.082346
16	H	3.881047	1.354736	–0.195792
17	H	–0.246551	3.839268	0.031959
18	H	–2.632751	3.169317	0.115853
19	H	3.611781	–1.562693	–1.270515
20	H	2.889815	–2.234600	0.198663
21	H	4.414526	–0.687085	1.264708

Table 9S. **A**(T_1) of CHQ-OAc

Center Number	Atom	Coordinates (Angstroms)		
		X	Y	Z
1	C	-1.111564	-0.492426	0.000137
2	C	0.241639	-0.042039	0.000091
3	C	0.473242	1.379998	-0.000024
4	C	-0.626100	2.293464	-0.000005
5	C	-1.911603	1.861115	0.000075
6	C	-2.249409	0.438662	0.000218
7	N	1.232889	-0.944974	0.000041
8	C	2.520700	-0.512779	-0.000089
9	C	2.831670	0.891125	-0.000138
10	C	1.817060	1.810958	-0.000156
11	C	3.525137	-1.483463	0.000197
12	O	-3.419281	0.045374	0.000058
13	Cl	-1.465183	-2.168036	-0.000182
14	H	4.571890	-1.208659	0.000482
15	H	3.252209	-2.529499	0.000565
16	H	3.869085	1.204080	-0.000221
17	H	2.031952	2.874240	-0.000268
18	H	-0.405754	3.356291	-0.000099
19	H	-2.751877	2.544285	0.000047

Sum of electronic and zero-point energies: -974.783608
Zero-point correction= 0.131184 (Hartree/Particle)

Table 10S. **T**(T_1) of CHQ-OAc

Center Number	Atom	Coordinates (Angstroms)		
		X	Y	Z
1	C	-2.019328	-0.822464	-0.100552
2	C	-1.171937	0.289030	-0.088563
3	C	-1.719639	1.581196	0.145556
4	C	-3.111258	1.710563	0.347786
5	C	-3.960276	0.628897	0.334763
6	C	-3.469830	-0.705569	0.113697
7	N	0.183283	0.157812	-0.304334
8	C	1.067852	1.225751	-0.276077
9	C	0.547193	2.497180	-0.027695
10	C	-0.800112	2.705381	0.175631
11	C	2.485368	0.932073	-0.602149
12	O	2.942190	-0.142787	0.273248
13	C	4.252065	-0.481237	0.150133
14	C	4.603904	-1.615408	1.078409
15	O	-4.202000	-1.714475	0.099059
16	Cl	-1.371298	-2.406664	-0.372711
17	O	5.005114	0.073468	-0.606821
18	H	-1.188943	3.696250	0.368286
19	H	1.237204	3.332720	-0.004814
20	H	-3.506807	2.705259	0.522177
21	H	-5.026230	0.731679	0.491728
22	H	2.617801	0.588228	-1.634895
23	H	3.108170	1.815388	-0.460985
24	H	5.668180	-1.826835	1.001961
25	H	4.343633	-1.352967	2.105799
26	H	4.029109	-2.505631	0.812266
27	H	0.560514	-0.777495	-0.364622

Sum of electronic and zero-point energies: -1203.885573
Zero-point correction= 0.196822 (Hartree/Particle)

Table 11S. **Z**(T_1) of CHQ-OAc

Center Number	Atomic Number	Coordinates (Angstroms)		
		X	Y	Z
1	C	1.817011	1.810790	0.000230
2	C	0.473316	1.379884	0.000085
3	C	0.241947	-0.042153	-0.000190
4	N	1.232899	-0.945269	-0.000237
5	C	2.520869	-0.513028	0.000186
6	C	2.831718	0.891000	0.000044
7	C	-0.626205	2.293490	-0.000007
8	C	-1.911605	1.861347	-0.000077
9	C	-2.249354	0.438811	0.000462
10	C	-1.111474	-0.492546	0.000036
11	Cl	-1.465490	-2.167970	0.000161
12	O	-3.419076	0.045500	-0.000414
13	C	3.525105	-1.483104	-0.000540
14	H	2.031993	2.874148	0.000107
15	H	3.869137	1.203933	-0.000021
16	H	-0.405795	3.356247	-0.000115
17	H	-2.752046	2.544254	-0.000326
18	H	3.252620	-2.529424	0.000735
19	H	4.571787	-1.207737	0.000484

Sum of electronic and zero-point energies: -974.793800
Zero-point correction= 0.131188 (Hartree/Particle)

Synthetic Procedures

8-Bromo-7-(methoxymethoxy)-2-methylquinoline (2). 8-Bromo-2-methylquinolin-7-ol (**1**, 1.83 g, 7.38 mmol) was dissolved in THF (30 mL). Triethylamine (2.00 mL, 14.2 mmol) was added to the solution. After 2 min of stirring, chloromethyl methyl ether (1.00 mL, 13.2 mmol) was added dropwise and the reaction was stirred overnight. The solvents were evaporated, and the residue dissolved with chloroform (100 mL). The chloroform solution was washed with water and brine, and dried over anhydrous Na_2SO_4. The solvent was evaporated and the residue was purified by flash chromatography with EtOAc/hexane (1:9) to yield **2** (1.74 g, 6.17 mmol, 84%). ^{1}H NMR (400 MHz, $CDCl_3$, Me_4Si) δ: 7.98 (1H, d, *J* = 8.0 Hz), 7.70 (1H, d, *J* = 9.2 Hz), 7.43 (1H, d, *J* = 9.2 Hz), 7.24 (1H, d, *J* = 8.0 Hz), 5.40 (2H, s), 3.58 (3H, s), 2.80 (3H, s). ^{13}C NMR (101 MHz, $CDCl_3$, Me_4Si) δ: 161.1, 155.1, 146.3, 136.5, 128.0, 123.6, 121.3, 116.6, 112.1, 95.6, 56.8, 26.1. FTIR (neat) 2906, 2844, 1614, 1508, 1322, 1248, 1154, 1056, 993, 920, 831 cm^{-1}. HR-MS (ESI): *m/z* calcd for $(C_{12}H_{12}BrNO_2+H)^+$ 282.0130 (^{79}Br) and 284.0109 (^{81}Br), found 282.0132 and 284.0107.

8-Bromo-7-(methoxymethoxy)quinoline-2-carbaldehyde (3). 8-Bromo-7-(methoxymethoxy)-2-methylquinoline (**2**, 250 mg, 0.887 mmol) was dissolved in *para*-dioxane (5 mL). Selenium dioxide (100 mg, 0.901 mmol) was added to the solution, and the mixture was heated at 80 °C for 4 h with stirring. The reaction mixture was vacuum filtered and the filtrate was concentrated. The remaining residue was purified by flash chromatography using EtOAc/hexane (1:3) to provide **3** (205 mg, 0.693 mmol, 78%). ^{1}H NMR (400 MHz, $CDCl_3$, Me_4Si) δ: 10.30 (1H, s), 8.27 (1H, d, *J* = 8.4 Hz), 7.97 (1H, d, *J* = 8.4 Hz), 7.84 (1H, d, *J* = 9.2 Hz), 7.66 (1H, d, *J* = 9.6 Hz), 5.45 (2H, s), 3.60 (3H, s). ^{13}C NMR (101 MHz, $CDCl_3$, Me_4Si) δ: 193.9, 156.1, 153.6, 146.5, 138.0, 128.2, 127.2, 119.9, 116.5, 113.3, 95.6, 57.0. FTIR (neat): 2933, 2834, 1708, 1615, 1442, 1261, 1156, 1038, 920, 839 cm^{-1}. HR-MS (ESI): *m/z* calcd for $(C_{12}H_{10}BrNO_3+H)^+$ 295.9922 (^{79}Br) and 297.9902 (^{81}Br), found 295.9925 and 297.9895.

(8-Bromo-7-(methoxymethoxy)quinolin-2-yl)methanol (4). 8-Bromo-7-(methoxymethoxy)-quinoline-2-carbaldehyde (**3**, 150 mg, 0.51 mmol) was mixed with ethanol, then sodium borohydride (15 mg, 0.41 mmol) was added in small portions to the mixture. When the reaction was complete as determined by TLC, it was concentrated, and the remaining residue was diluted with chloroform. The mixture was then washed successively with water and brine, dried over anhydrous Na_2SO_4, and evaporated to provide **4** (140 mg, 0.47 mmol, 92%). ^{1}H NMR (400 MHz, $CDCl_3$, Me_4Si) δ: 8.08 (1H, d, *J* = 8.4 Hz), 7.76 (1H, d, *J* = 8.8 Hz), 7.50 (1H, d, *J* = 8.8 Hz), 7.22 (1H, d, *J* = 8.4 Hz), 5.42 (2H, s), 4.94 (2H, s), 3.58 (3H, s). ^{13}C NMR (101 MHz, $CDCl_3$, Me_4Si) δ: 160.5, 155.6, 145.0, 137.2, 128.1, 124.5, 117.4, 117.1, 112.1, 95.6, 64.3, 56.9. FTIR (neat): 3294, 2935, 1618, 1514, 1256, 1197, 1152, 1055, 989, 916, 835 cm^{-1}. HR-MS (ESI): *m/z* calcd for $(C_{12}H_{12}BrNO_3+H)^+$ 298.0079 (^{79}Br) and 300.0058 (^{81}Br), found 298.0075 and 300.0056.

(8-Bromo-7-(methoxymethoxy)quinolin-2-yl)methyl acetate (5). (8-Bromo-7-(methoxymethoxy)quinolin-2-yl)methanol (**4**, 0.05 g, 0.17 mmol) was dissolved in a small amount of chloroform. Acetic anhydride (0.1 mL), pyridine (0.5 mL), and DMAP (0.01 g) were added and the reaction stirred overnight. The reaction was concentrated and the remaining residue purified by column chromatography with a gradient from 100% hexanes to 1:1 EtOAc/Hex (0.055 g, 95%) to provide **5**. ^{1}H NMR (400 MHz, $CDCl_3$, Me_4Si) δ: 8.12 (d, *J* = 8.4 Hz, 1H), 7.75 (d, *J* = 9.0 Hz, 1H), 7.50 (d, *J* = 9.0 Hz, 1H), 7.40 (d, *J* = 8.4 Hz, 1H), 5.47 (s, 2H), 5.41 (s, 2H), 3.58 (s, 3H), 2.24 (s, 3H). ^{13}C NMR (101 MHz, $CDCl_3$, Me_4Si) δ:

170.9, 157.9, 155.5, 154.5, 147.1, 137.2, 128.0, 124.6, 123.8, 117.6, 95.7, 67.4, 56.8, 21.2. HR-MS (ESI): *m/z* calcd for $(C_{14}H_{14}BrNO_4+H)^+$ 340.0184 (^{79}Br) and 342.0164 (^{81}Br), found 340.0175and 342.0157.

(8-Bromo-7-hydroxyquinolin-2-yl)methyl acetate (BHQ-OAc). (8-Bromo-7-(methoxy-methoxy)quinolin-2-yl)methyl acetate (**5**, 0.055 g, 0.16 mmol) was dissolved in chloroform. Trifluoroacetic acid was added and the reaction stirred for 2 h. The reaction was transferred to a separatory funnel and neutralized with 5% sodium bicarbonate. The combined organic layers were washed with brine, dried over $MgSO_4$, filtered, and concentrated. The crude residue was purified by HPLC with 40% CH_3CN/60% H_2O (0.1% TFA) to provide BHQ-OAc (0.035 g, 75%). This material was spectroscopically identical to the data given in the literature.[5]

(7-hydroxyquinolin-2-yl)methyl acetate (HQ-OAc). Tetrabutyl ammonium fluoride (0.18 mL, 1 M in THF) was added to a solution of (7-((*tert*-butyldiphenylsilyl)oxy)quinolin-2-yl)methyl acetate (**6**, 0.081 g, 0.18 mmol) in THF (1 mL). The resulting solution was stirred at room temperature for 1.5 h before diluting with EtOAc, washing with water, drying over $MgSO_4$, and filtering. The solvent was evaporated and the remaining residue purified by flash chromatography with EtOAc/hexane (7:3) to yield HQ-OAc (0.026 mg, 0.12 mmol, 67%). ^{1}H NMR (400 MHz, $CDCl_3$, Me_4Si) δ: 7.93 (1H, d, J = 8.4 Hz), 7.28 (1H, s), 7.20 (2H, d, J = 8.4 Hz), 6.73 (1H, d, J = 8.4 Hz), 5.28 (2H, s), 2.05 (3H, s). ^{13}C NMR (101 MHz, $CDCl_3$, Me_4Si) δ: 170.9, 159.5, 155.6, 148.2, 137.5, 128.9, 122.1, 120.0, 116.7, 108.6, 103.5, 66.5, 21.0. HR-MS (ESI): *m/z* calcd for $(C_{12}H_{11}NO_3+H)^+$ 218.0817, found 218.0818.

References

[1] M. J. Frisch, G. W. Trucks, H. B. Schlegel, G. E. Scuseria, M. A. Rob, J. R. Cheeseman, J. A. Montgomery, Jr., T. Vreven, K. N. Kudin, J. C. Burant, J. M. Millam, S. S. Iyengar, J. Tomasi, V. Barone, B. Mennucci, M. Cossi, G. Scalmani, N. Rega, G. A. Petersson, H. Nakatsuji, M. Hada, M. Ehara, K. Toyota, R. Fukuda, J. Hasegawa, M. Ishida, T. Nakajima, Y. Honda, O. Kitao, H. Nakai, M. Klene, X. Li, J. E. Knox, H. P. Hratchian, J. B. Cross, V. Bakken, C. Adamo, J. Jaramillo, R. Gomperts, R. E. Stratmann, O. Yazyev, A. J. Austin, R. Cammi, C. Pomelli, J. W. Ochterski, P. Y. Ayala, K. Morokuma, G. A. Voth, P. Salvador, J. J. Dannenberg, V. G. Zakrzewski, S. Dapprich, A. D. Daniels, M. C. Strain, O. Farkas, D. K. Malick, A. D. Rabuck, K. Raghavachari, J. B. Foresman, J. V. Ortiz, Q. Cui, A. G. Baboul, S. Clifford, J. Cioslowski, B. B. Stefanov, G. Liu, A. Liashenko, P. Piskorz, I. Komaromi, R. L. Martin, D. J. Fox, T. Keith, M. A. Al-Laham, C. Y. Peng, A. Nanayakkara, M. Challacombe, P. M. W. Gill, B. Johnson, W. Chen, M. W. Wong, C. Gonzalez, J. A. Pople, Revision C.02 ed., Gaussian, Inc., Wallingford, CT, **2003**.

[2] Y. Zhu, C. M. Pavlos, J. P. Toscano, T. M. Dore, *J. Am. Chem. Soc.* **2006**, *128*, 4267-4276.

[3] a) D.-Y. Hwang, A. M. Mebel, *J. Phys. Chem. A* **2002**, *106*, 520-528; b) B. Maiti, G. C. Schatz, *J. Chem. Phys.* **2003**, *119*, 12360-12371.

[4] C. Ma, Y. Du, W. M. Kwok, D. L. Phillips, *Chem.—Eur. J.* **2007**, *13*, 2290-2305.

[5] O. D. Fedoryak, T. M. Dore, *Org. Lett.* **2002**, *4*, 3419-3422.
